# Variation in the stringency of COVID-19 public health measures on self-reported health, stress, and overall wellbeing in Canada

**DOI:** 10.1038/s41598-023-39004-w

**Published:** 2023-08-11

**Authors:** Emily Cameron-Blake, Henry Annan, Leonora Marro, David Michaud, Julia Sawatzky, Helen Tatlow

**Affiliations:** 1 Blavatnik School of Government, University of Oxford, Radcliffe Observatory Quarter, Woodstock Road, Oxford, OX2 6GG UK; 2 School of Social and Political Science, University of Edinburgh, 15a George Square, Edinburgh, EH8 9LD UK; 3 Faculty of Medicine, Dalhousie University, Halifax, NS Canada; 4 IWK Health, Halifax, NS Canada; 5 Health Canada, Environmental and Radiation Health Sciences Directorate, Environmental Health Science and Research Bureau, Biostatistics Section, 251 Sir Frederick Banting Driveway, Tunney’s Pasture, Ottawa, ON K1A0K9 Canada; 6 Health Canada, Environmental and Radiation Health Sciences Directorate, Consumer and Clinical Radiation Protection Bureau, 775 Brookfield Road, Ottawa, ON K1A 1C1 Canada; 7 Faculty of Medicine and Dentistry, University of Alberta, Edmonton, Canada

**Keywords:** Infectious diseases, Health policy

## Abstract

Evidence is building regarding the association between government implemented public health measures aimed at combating COVID-19 and their impacts on health. This study investigated the relationship between the stringency of public health measures implemented in Canada and self-reported mental health, physical health, stress, and wellbeing among a random sample of 6647 Canadians 18 years of age and older. The analysis was based on self-reported health data from the *Canadian Perspectives on Environmental Noise Survey*. This data was combined with the Oxford COVID-19 Government Response Tracker database, which included overall stringency index (SI), and four of its sub-components, i.e., school and business closures, restrictions on gatherings, and stay at home policies. Adjusted multivariate logistic regression models indicated that the magnitude of the overall SI was associated with higher or lower odds of reporting worse physical health, mental health, stress and/or overall wellbeing, depending on the measure evaluated. Similarly, policy directed at the four sub-components had varying impacts on the odds of reporting worse health, depending on the sub-component, the strength of the policy restriction, and the health outcome evaluated. The association between the strength of the public health measures and self-reported health, and how this may inform future policy, is discussed.

## Introduction

It comes as no surprise that the severe acute respiratory syndrome coronavirus 2 (SARS-CoV-2, hereinafter COVID-19) pandemic has indirectly and adversely affected perceived health and stress in Canada^1^. Crowe et al.^2^ reported increased incidences of post-traumatic stress disorder (PTSD), depression and anxiety in critical care nurses in Canada (both PTSD and anxiety measured using self-reported validation surveys—Impact of Events Scale-Revised [IES-R] and the Depression, Anxiety and Stress Scale [DASS-21] respectively) due in part to rapidly changing policies, at times unclear and overwhelming information, and the stress of managing patient needs and safety with home life. A statistical association was observed between periods of COVID-19 quarantine and risk of self-reported poor mental health and increased risk of suicidal ideation^3^. Studies by Samji et al.^4,5^ suggested that children and adolescents in Canada and around the world were experiencing more self-reported depression and anxiety symptoms than pre-pandemic times, potentially resulting from less interpersonal interactions, lack of outdoor time, increased tensions within families, worry about school, struggling with the transition to online learning, and increased media consumption regarding COVID-19. These findings are not unique to Canada—a study from Italy found increased rates of PTSD, depression, and anxiety in healthcare workers during the COVID-19 pandemic^6^. Wang et al.^7^ reported that 53.8% of general public respondents in China rated the psychological impact of the pandemic as moderate or severe. Lockdowns due to COVID-19 were associated with higher prevalence of PTSD in male graduate students in China using the IES-R scale^8^, while lockdowns on German universities were associated with increased symptoms of depression and loneliness on the Patient Health Questionnaire-9^9^.

In recognition of the strain the pandemic was having on Canadians, just as the third wave of the pandemic was rolling through Canada (25 Feb 2021 onwards), regular statements, and updates from the Chief Public Health Officer (CPHO) of Canada were all accompanied with a statement about mental health and where Canadians could access support^10^. The 2020 CPHO annual report^11^ indicated that the initial waves of COVID-19 had a significant impact on the mental health of Canadians: in 2018, 66% of Canadians aged 15 years and older self-reported excellent/very good mental health, which decreased to 48% in early May 2020. The 2021 CPHO annual report^12^ showed the same trend of worsening mental health and wellness during the pandemic with 42% of Canadians indicating that their self-perceived mental health was “somewhat worse” or “much worse” when compared to before the pandemic. These results were more common in women (44%) compared to men (39%). The ways in which the pandemic and the implementation of public health measures may have affected the mental and physical health of Canadians is poorly understood yet could inform the development of appropriate policies and strength of interventions should they be required in the future.

The *Canadian Perspectives on Environmental Noise Survey* (CPENS), conducted by Health Canada between April 12, 2021, and May 25, 2021, investigated attitudes, expectations, and perceptions of environmental noise in rural and non-rural Canada^13,14^. Because this period coincided with the third wave of the COVID-19 global pandemic, the potential influence that the pandemic may have had on survey respondents was considered. Beyond its primary focus on environmental noise, CPENS included content on the perceived impact that COVID-19 had on self-reported mental health, physical health, stress in life, and overall wellbeing. Depending on the outcome evaluated, between 43 and 67% of the respondents reported the measure as somewhat or much worse due to the pandemic^14^. What remains unclear is the extent to which these elements may have been impacted by public health and safety measures that limited human interactions and regular daily activities in order to prevent transmission of COVID-19.

**Figure 1:**
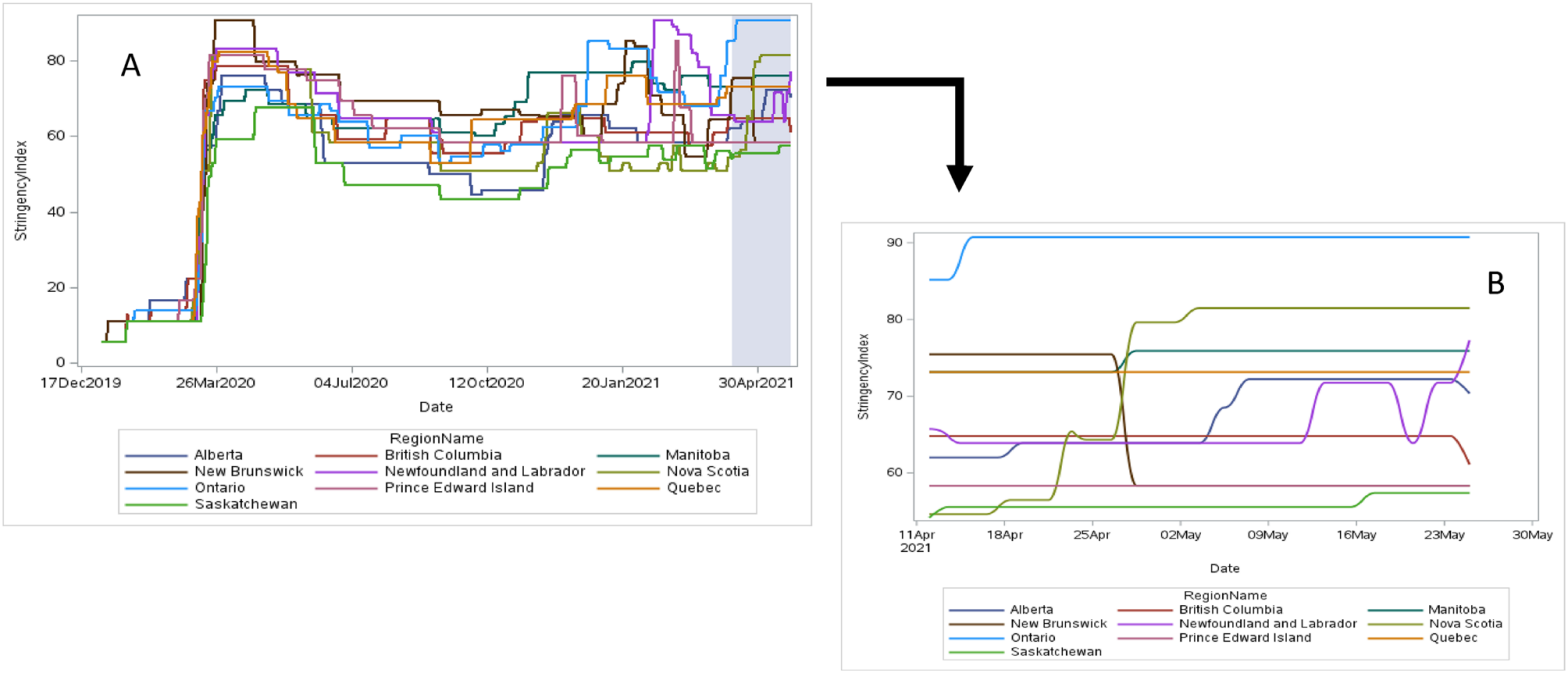
Stringency index by date in each province across Canada from January 1, 2020, to May 25, 2021 (Panel **A**) highlighting the survey period (April 12, 2021–May 25, 2021). Stringency index by date in each province across Canada during the survey period April 12, 2021–May 25, 2021 (Panel **B**).

During the CPENS data collection period, the pandemic experience and public health interventions and restrictions across Canada resembled global public health measures, yet varied from province to province (Table 1, Fig. 1).

**Table 1:** The provinces of Canada employed differing public safety measures during the COVID-19 pandemic.

Province	School closures	Stay-at-home orders	Business/workplace closures	Restrictions on gatherings	Travel restrictions
British Columbia (BC)			Restaurants/pubs/bars	No indoor gatherings permitted, max of 10 persons	
Alberta (AB)	Primary and secondary in targeted regions, then all schools (5 May)		Personal services, in-person dining	No indoor gatherings permitted, max of 10 persons	
Saskatchewan (SK)	Closures in targeted regions		Restaurants in targeted regions	No indoor gatherings permitted, households only	
Manitoba (MB)	Primary and secondary in targeted regions	Targeted regions	Non-essential closures in targeted regions	Indoor public gatherings limited to max of 5 persons	14-days quarantine upon entering province
Ontario (ON)	All schools/levels	Entire province	All non-essential	No gatherings permitted	Only essential movement allowed within province
Quebec (QC)	Closures in targeted regions	Province under curfew (8:00 pm to 5:00 am)	Social distancing and capacity limits in targeted regions; gyms closed	Varying restrictions in targeted regions	Restricted between targeted regions
New Brunswick (NB)	Closures in targeted regions	Targeted regions until 26 April	Targeted regions close non-essential until 26 April	Varying restrictions in targeted regions	14-days quarantine upon entering province, and restricted between targeted regions
Nova Scotia (NS)	Targeted regions, then all schools (28 April)		All non-essential closed from 28 April	Gatherings limited to max of 10 persons	14-days quarantine upon entering province, and restricted between targeted regions
Prince Edward Island (PE)			Limits on non-essential business capacity	Gatherings of maximum 50 persons permitted	14-days quarantine upon entering province
Newfoundland and Labrador (NL)	Highschool students on alternating days schedule	Targeted regions from mid-May	Targeted region closures, in-person dining and fitness venues	Varying restrictions in targeted regions	14-days quarantine upon entering province

Shown here are the strictest variations of five public safety measures taken by the 10 provinces during the CPENS survey period 12 April–26 May 2021. Where COVID-19 cases were greater in localised areas of a province, public safety measures may have applied only to these targeted regions. (Source, Oxford COVID-19 Government Response Tracker).

A recent study by Aknin et al.^15^ has evaluated policy stringency and mental health in 15 countries (Canada included) using the Oxford COVID-19 Government Response Tracker (OxCGRT) data, finding that higher stringency was associated with higher mean psychological distress scores, especially in countries that used a mitigating strategy (targeted policies to prevent widespread transmission of COVID-19) vs elimination (maximum policies aimed to eliminate COVID-19) strategy for battling COVID-19. Aknin et al*.* showed that increased psychological distress and lower life evaluation were associated with specific public health measures such as restrictions on gatherings and stay-at-home requirements, while school and workplace closures appeared to have no association with psychological distress or life evaluation. Variation in government responses has been documented using the OxCGRT stringency index (SI) and has been used elsewhere in relation to COVID-19^16–18^, demonstrating the usefulness of the SI as a tool for comparison across countries and jurisdictions. Recently, Plett et al.^19^, using the OxCGRT Canadian subnational data, have shown that mortality rates and stay-at-home measures in Canadian provinces were associated with increased anxiety.

The objective of this exploratory study was to investigate how the variation in stringency of multiple government-mandated public health measures may have been associated with the evaluated health outcomes reported in CPENS.

## Methods

### Sample

#### Target population, sample size and response rate

A detailed description of CPENS methodology and sample has been reported by Michaud et al.^13,14^. Essential details from these publications have been reproduced below. Briefly, a population probability-based random sample (GPRS) in all provinces was used to recruit respondents via telephone to the online survey. The GPRS is a proprietary representative sample source recruited via probability sampling. Non-respondents were sent a reminder message at 3 and 6 days after the initial recruitment. Between April 12, 2021, and May 19, 2021, a total of 24,133 phone numbers were called. Of the 22, 892 potentially eligible participants, 11,492 were recruited to the survey and 6647 completed the online survey, for an overall response rate among eligible respondents of 29.0%. The margin of error for the study was ± 1.2%, at a 95% confidence level.

#### Determining geographic sampling regions

The sampling frame was set to target respondents from remote/rural (less than 1000 inhabitants and between 1000 and 10,000 inhabitants respectively), suburban (mixed-use or residential area) and urban (city with more than 10,000 inhabitants) areas in all ten Canadian provinces using the forward sortation area (FSA) postal code information^20^. Respondents indicated the geographic region that best corresponded to the area in which they lived based on population size. Because some postal codes can be both rural and urban, geographic region in the statistical analysis was based on self-reported geographic region as either remote/rural, suburban, or urban^13,14^.

#### Questionnaire development, pretesting and quality control

The questionnaire for CPENS was designed by Health Canada and pre-tested in both English and French. For the pretesting, 299 people were recruited by phone (212 in English and 87 in French). This led to 72 completed online surveys (61 English, and 11 French). Minor changes made to the survey after pre-testing did not affect that pre-test data, and therefore results collected during the pre-test were included in the final analysis^13,14^.

The survey is available in English and French through Library and Archives Canada^21^. Although the survey content was intended to evaluate noise perception, annoyance, and expectations of quiet, the focus of the current analysis is on the assessment of the pandemic and stringency of public health measures on self-reported measures of health in CPENS. The average length of time to complete the online survey was just under 10 min. This study was approved by the Health Canada and Public Health Agency of Canada Review Ethics Board (Protocol no. REB 2020-038H). Informed consent is implied in the voluntary response to the survey questionnaire. This research was conducted in accordance with all relevant Government of Canada guidelines and regulations for conducting online surveys.

### Variable definitions

In CPENS, participants were asked to “indicate how they have been personally affected by the COVID-19 pandemic” with respect to physical health, mental health, stress in life, and overall wellbeing. Response categories for these four outcome variables were as follows: Much worse, somewhat worse, unchanged, somewhat improved, and much improved. For modelling, the responses were grouped into the two following categories “somewhat/much worse” and “unchanged/somewhat/much improved” (renamed as “unchanged/improved”). This grouping was required due to insufficient participants in some response categories. Separate sensitivity analyses were also carried out with the three categories “much worse”, “somewhat worse” and “unchanged/improved”.

A number of other variables were collected in CPENS that were considered to be potentially associated to the above four health-related outcome variables affected by COVID-19^13,14^. These included the demographic variables such as age, gender, education, income, and Indigenous status. Age in years were divided and collapsed into three groups (18–34, 35–54, 55+) due to low responses in some age categories. The following gender categories were defined (female, male, other/prefer not to say). Education was rated as: up to high school diploma or equivalent, certificate or diploma, bachelor's degree, or post graduate degree. A certificate or diploma could be from a registered apprenticeship, or other trade, college, CEGEP (i.e., Quebec College) or other non-university, university below bachelor's level. Total household income was grouped in Canadian dollars as follows: under $40 K, $40 K to just under $80 K, $80 K to just under $150 K, $150 K and above. Indigenous status was grouped as follows: “Self-identify as First Nation/Métis/Inuk (Inuit)”, or “Do not self-identify as an Indigenous person”. Province of residence as well as geographic location were also considered as potential predictor variables since the response to the pandemic differed by province as well as geographic location^13,14^.

A respondent’s current work or school situation was also considered. Respondents self-identified as follows: working or attending school outside their home; working or attending school inside their home; retired; unemployed; and a portion of those indicating “other” could be grouped as on paid leave (sick, maternity, and disability). More than one option could be selected; therefore, each situation was considered separately as a “Yes/No” response.

Overall physical health, mental health and anxiety/depression were investigated for their association to the health-related outcome variables affected by the COVID-19 pandemic. Participants were asked to rate their overall physical health and mental health relative to someone of their age, prior to questions related to COVID-19, in order to gauge their general health status. For both of these questions the responses included the following: poor; fair; good; very good; and excellent. These were collapsed into two groups: poor/fair and good/very good/excellent, an intuitive grouping of negative and positive outcomes, and due to low numbers of respondents for some responses. Anxiety or depression, was evaluated as diagnosed by a healthcare professional, not diagnosed but suffer from the condition, or does not apply.

The models were also adjusted for the provincial rates of cases and deaths due to COVID-19 in the 7-day rolling average prior to each respondent’s survey completion date and the province of residence. Rates are given per 100,000 people of the population. The data was extracted from the Government of Canada COVID-19 epidemiology updates^22^. Rate of deaths and cases for the past 7 days is recorded for each day in the province by provincial health authorities. The rate of deaths/cases was linked to participants’ in CPENS by completion date of their survey and province of residence. No further grouping of this variable was applied.

### COVID-19 stringency index

The OxCGRT systematically records government policies on 24 areas of COVID-19 protection and prevention in 185 countries, and subnational data on the provinces and territories of Canada in a longitudinal panel dataset beginning January 1, 2020, until 31 December 2022^23,24^. Combinations of these policy indicators are aggregated into several indices including the SI, which reflects the overall strength of closure and containment measures of a jurisdiction into a score between 0 (lowest stringency) and 100 (highest stringency). The SI includes 9 indicators (sub-components): school closures, business/workplace closures, cancellation of public events, restrictions on gatherings, public transport restrictions, stay-at-home orders, restrictions on internal (domestic) movement, international travel restrictions and public health campaigns. Full methodology of the SI calculations can be found in Hale et al.^23^.

**Figure 2:**
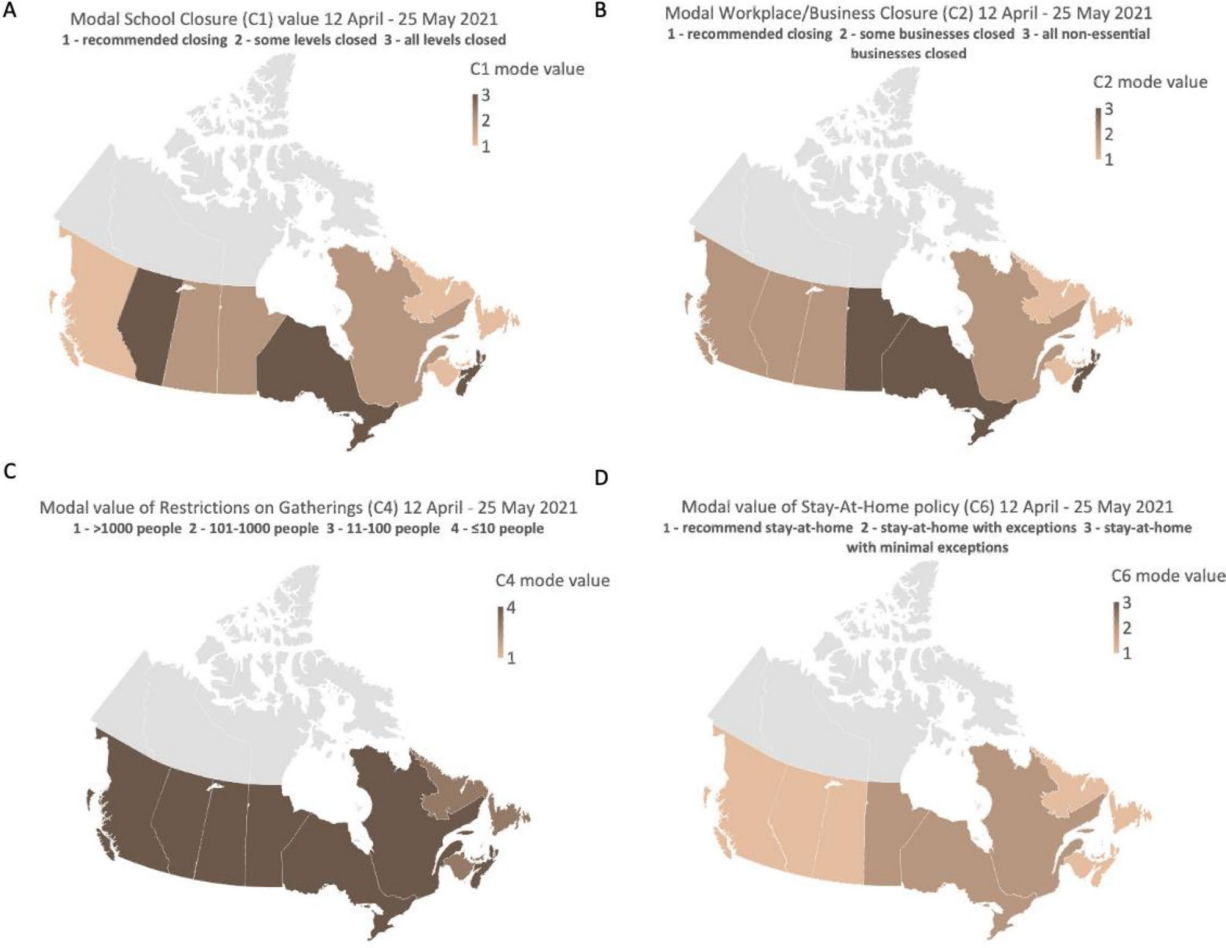
A graphical representation of the variation in government-mandated public health measures across all Canadian provinces. The maps show the most frequently occurring (mode) restriction level for school closures (Panel **A**), workplace/business closures (Panel **B**), restrictions on gatherings (Panel **C**) and stay at home orders (Panel **D**) during the survey sampling period (April 12–May 25, 2021). The Territories of Canada were not included in CPENS due to low populations density. (Map created using OxCGRT data in Excel for Microsoft 365 v.16).

Canadian subnational data includes measures taken by the provincial/territorial level of government for all indicators except for international travel (determined by the federal government). Every indicator of the SI is measured on an ordinal scale (varying from 0–2 up to 0–4, indicator dependent) (see Fig. 2 for description of each ordinal level), with each increment representing a more stringent policy. The OxCGRT records the strictest policy in place for a province, and a binary flag that represents whether it is applicable to the entire jurisdiction (general) or to a specific region within that jurisdiction (targeted). For the current study, we evaluated magnitude of the SI and severity among four of the nine sub-components for each province (i.e., school closures, business/workplace closures, restrictions on gatherings and stay-at-home orders), to explore associations between these policies and the aforementioned self-reported health outcomes.

For each participant in the survey their overall SI value was set equal to the SI that corresponded to the date the participant completed the survey and their province. To achieve sufficient sample sizes, the SI was grouped into 10-unit increments (50 to < 60, 60 to < 70, 70 to < 80, and 80+). Groups of 5 increments of SI were considered (i.e., 50 to < 55), but the associated sample sizes were too small to conduct meaningful analyses.

The SI was considered and compared to the prevalence of reporting “somewhat/much worse” for each of the four health-related outcomes reported to be affected by COVID-19. The mode of the SI was calculated in two different manners: (1) During the period that the survey was conducted (April 12–May 25, 2021); and 2) From the start of the pandemic in Canada (March 13, 2020) to the conclusion of the survey (May 25, 2021). When linking SI to each respondent’s province and date of response, the lowest SI observed during the CPENS data collection period was 54.17. The mode, shown in Fig. 2, represents the most repeated level of restriction in each province during the COVID-19 pandemic over the survey data collection period (i.e., April 12–May 25, 2021) for school closures (panel A), workplace/business closures (panel B), restrictions on gatherings (panel C), and stay-at-home policies (panel D). The mode was preferable as the mean was highly influenced by minimum and maximum values without representing what would otherwise be the most experienced levels of restrictions during the pandemic. Likewise, the mode was used in place of the maximum SI value, since the maximum would set every province to the worst-case during the pandemic preventing an assessment of how the variation in these measures may have affected the evaluated outcomes.

### Statistical methodology

Weighted frequencies and cross-tabulations were used to explore the distribution of each health-related outcome reported to be affected by COVID-19 across SI and the four subcomponents (business/workplace closure, school closure, restrictions on gatherings and stay-at-home orders sub-ratings). The data was weighted to match the marginal population proportions for age, gender, Indigenous status, and geographic location^13,14^. Univariate logistic regression models were used to assess the association between SI/subcomponents with health-related outcomes. When the overall Wald chi-square from logistic regression $(\chi^{2} )$ was significant then post hoc pairwise tests with Bonferroni adjustment were applied to control for the overall type I error rate to be less than 0.05.

Multivariate logistic regression models were used to assess the relationship between the prevalence of reporting “somewhat/much worse” in each of the health-related outcomes and the aforementioned SI groups, as well as the business/workplace closure, school closure, restrictions on gatherings and stay-at-home orders sub-components. In addition to adjusting all models for age, gender, Indigenous status, geographical location, income, work status, general physical and mental health, anxiety/depression, models were also adjusted for the rate of death due to COVID-19 and rate of COVID-19 cases per 100,000, controlling for the intensity of the pandemic. These variables were included in the model to improve precision of determining if COVID-19 restrictions were associated with the outcome variable. Province of residence was used in the descriptive analysis, but not in the multivariate logistic regression model as province was highly correlated with SI groups, business/workplace closure, school closure, restrictions on gatherings and stay-at-home orders. Adjusted odds ratios (ORs) are reported for each relationship. All ORs are compared to the reference category of the variable as listed in the tables and figures (see Supplemental Tables S1–S5 for additional OR values). Income and education were highly associated; to avoid issues of collinearity only income was retained in the models.

Multinomial regression models were used to model the incidence of selecting “much worse” or “somewhat worse” versus the reference category “unchanged/improved” for each of the four COVID-19 related health outcome variables (physical health, mental health, stress in life, and overall wellbeing). This sensitivity analysis was applied to investigate the differences of modelling the outcome “somewhat/much worse” in a logistic regression versus modelling the outcomes “much worse” or “somewhat worse” in a multinomial regression model.

Statistical analysis was performed using SAS Enterprise Guide 7.15 (SAS Institute Inc., Cary, NC). All regression models are based on the weighted data. A 0.05 statistical significance level was implemented throughout unless otherwise stated. In addition, Bonferroni corrections were made to account for all pairwise comparisons to ensure that the overall Type I (false positive) error rate was less than 0.05. Hosmer–Lemeshow goodness of fit test, which assesses whether or not the observed event rates match the expected event rates in subgroups of the model population, is reported for all tests. A p-value greater than 0.05 indicates that the model fits the data well. When the p-value of the Hosmer–Lemeshow test is less than 0.05 then caution must be used in interpreting the results as the model does not fit the data well.

## Results

### Sample characteristics

A detailed description of the sample characteristics has been previously published^13^. Briefly, most CPENS respondents were 55 + years old (38.6%) followed by 35- to 54-year-olds (34.1%) and 18- to 34-year-olds (27.3%). Males (48.0%) and females (50.6%) were equally represented in the sample, while 1.3% of respondents identified as ‘other’ or ‘prefer not to say’. Most participants reported as having either a bachelor or post graduate degree (44.0%); and having a household income of $80 to < $150 K (35.9%) followed by $40 to < $80 K (28.0%). The majority of participants were from Ontario (40.3%) followed by Quebec (18.6%) and BC (14.4%).

### Preliminary analysis of COVID-19 outcomes with stringency index and its sub-components

**Figure 3:**
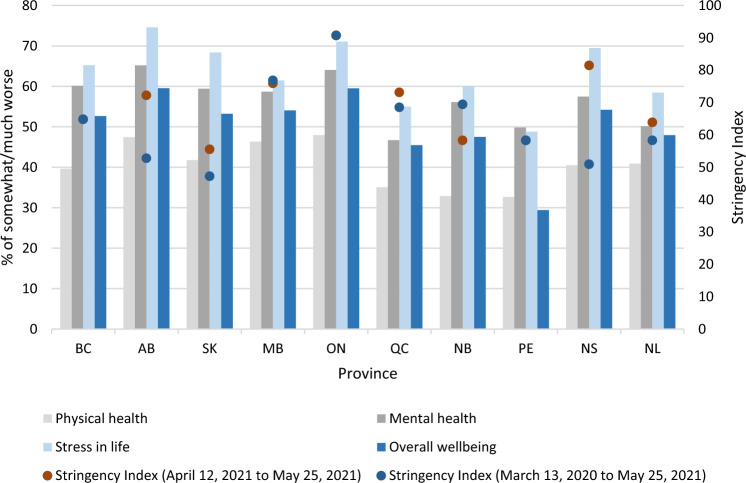
Reported impact of COVID-19 on health-related outcomes with stringency index. Bars represent the prevalence of people who reported “somewhat/much worse” in each of the COVID-19 health related outcomes. Where only one dot is visible, the two modal Stringency Index periods are the same or similar. BC—British Columbia, AB—Alberta, SK—Saskatchewan, MB—Manitoba, ON—Ontario, QC—Quebec, NB—New Brunswick, PE—Prince Edward Island, NS—Nova Scotia, NL—Newfoundland and Labrador.

The overall prevalence of feeling “somewhat/much worse” in each of the four health-related outcomes reported to be impacted by COVID-19 are graphically shown by province in Fig. 3. Generally, in provinces, the reported impact of COVID-19 was highest on stress in life, followed by their mental health, overall wellbeing, and physical health. The highest prevalence rates of these outcomes were observed in Alberta, Ontario, Saskatchewan, and Nova Scotia. The mode of the SI both during CPENS data collection (red dot) and in the previous year (blue dot) are also presented in Fig. 3; where a single dot appears, the two values overlap. In most provinces, the SI was similar during the previous year when compared to the time the survey was conducted, except in Alberta and Nova Scotia where the SI was higher during data collection. It was observed that provinces with a higher mode of SI (either during the survey or over the previous year) generally had a higher prevalence of reporting “somewhat/much worse” with respect to stress in life, overall wellbeing, and mental health. Notable exceptions to this were in Saskatchewan, which had relatively low SI modes, yet a relatively high prevalence of reporting somewhat/much worse. Furthermore, although the SI mode in and Saskatchewan and Prince Edward Island were similar, they differed on the prevalence of reporting somewhat/much worse in stress in life, overall wellbeing, and mental health.

**Figure 4:**
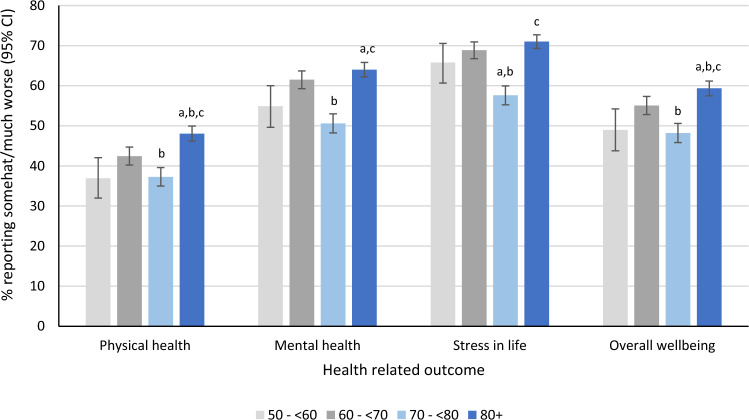
Prevalence (and its 95% confidence interval) of reporting “somewhat/much worse” in the four-health related COVID-19 outcomes (physical health, mental health, stress in life and overall wellbeing) by stringency index group (results obtained from univariate logistic regression models). The overall Wald chi-square from logistic regression $(\chi^{2} )$ was significant for all health-related outcomes (physical health $\chi_{3}^{2}$ = 56.31, *p* < 0.001; mental health $\chi_{3}^{2}$ = 83.78, *p* < 0.001; stress in life $\chi_{3}^{2}$ = 88.86, *p* < 0.001; overall wellbeing $\chi_{3}^{2}$ = 56.87, *p* < 0.001). Post hoc pairwise comparisons with Bonferroni adjustments are presented above with the following notations: ^a^significantly different (*p* < 0.05) compared to stringency index group 50 to < 60; ^b^significantly different (*p* < 0.05) compared to stringency index group 60 to < 70; ^c^significantly different (*p* < 0.05) compared to stringency index group 70 to < 80.

Figure 4 presents the prevalence rates of reporting “somewhat/much worse” in each of the health-related outcomes by SI group. All pairwise comparisons of SI groups applied Bonferroni corrections for multiple comparisons. For physical health ($\chi_{3}^{2}$ = 56.31, *p* < 0.001); SI group 60 to < 70 was higher compared to SI group 70 to < 80, *p* = 0.009, and SI group 80 + was higher compared to SI groups 50 to < 60, *p* < 0.001, 60 to < 70, *p* = 0.001, and 70 to < 80, *p* < 0.001. For mental health ($\chi_{3}^{2}$ = 83.78, *p* < 0.001); SI group 60 to < 70 was higher when compared to SI group 70 to < 80, *p* < 0.001, and SI group 80 + was higher compared to the SI groups 50 to < 60, *p* = 0.005, and 70 to < 80, *p* < 0.001. For stress in life ($\chi_{3}^{2}$ = 88.86, *p* < 0.001); SI group 70 to < 80 was decreased compared to both SI groups 50 to < 60, *p* = 0.025, and 60 to < 70, *p* < 0.001, and SI group 80 + was higher compared to the SI group 70 to < 80, *p* < 0.001. Finally, for overall wellbeing ($\chi_{3}^{2}$ = 56.87, *p* < 0.001); SI group 60 to < 70 was elevated compared to 70 to < 80, *p* < 0.001, and SI group 80 + was higher compared to SI groups 50 to < 60, *p* = 0.001, 60 to < 70, *p* = 0.025, and 70 to < 80, *p* < 0.001.

**Figure 5:**
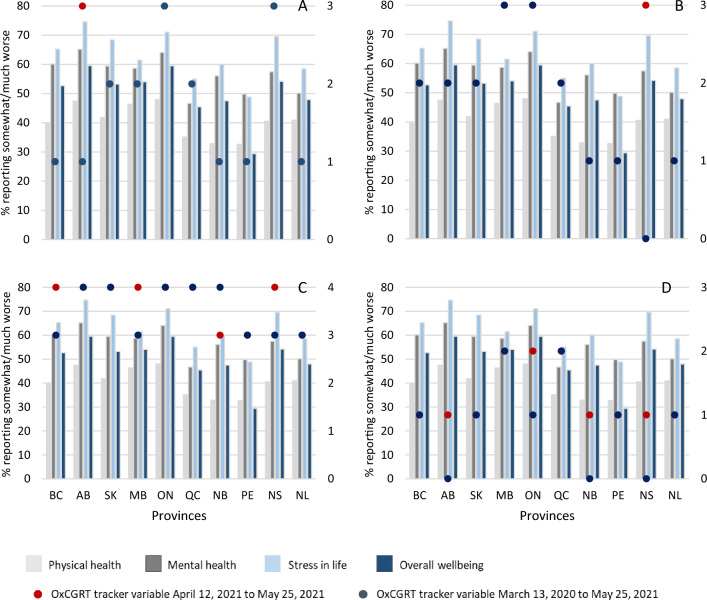
Reported impact of COVID-19 on health-related outcomes with (Panel **A**): school closures, (Panel **B**): business/workplace closures, (Panel **C**): restrictions on gatherings and (Panel **D**): stay-at-home orders. Bars represent the prevalence of people who reported feeling “somewhat/much worse” in each of the COVID-19 health related outcomes. Where only one dot is visible, the restrictions of the two periods are the same or similar. Right axis corresponds to the ratings of the OxCGRT variables. BC—British Columbia, AB—Alberta, SK—Saskatchewan, MB—Manitoba, ON—Ontario, QC—Quebec, NB—New Brunswick, PE—Prince Edward Island, NS—Nova Scotia, NL—Newfoundland and Labrador.

The prevalence rates of those feeling “somewhat/much worse” in each of the health-related outcomes by school closure, business/workplace closures, restrictions on gatherings and stay-at-home orders, respectively, are shown in Fig. 5, in each province. The ratings for these four variables ranged from 0 to 3 (0–4 for restrictions on gatherings), with 3 (4 for restrictions on gatherings) being the strictest rating. For the variable “School closure”, there was no rating 0 for the mode; for the variable “Stay-at-home orders”, there was no rating 0 or 3 for the mode. The mode on restrictions on gatherings was either 3 or 4.

Supplemental tables S6-S10 present the prevalence rates of feeling “somewhat/much worse”, “unchanged” and “somewhat/much improved” in each of the health-related outcomes by school closure, business/workplace closures, restrictions on gatherings, stay-at-home orders, and SI, along with sample sizes in subcomponents and SI.

### Multivariate logistic regression on all COVID-19 outcomes

**Figure 6:**
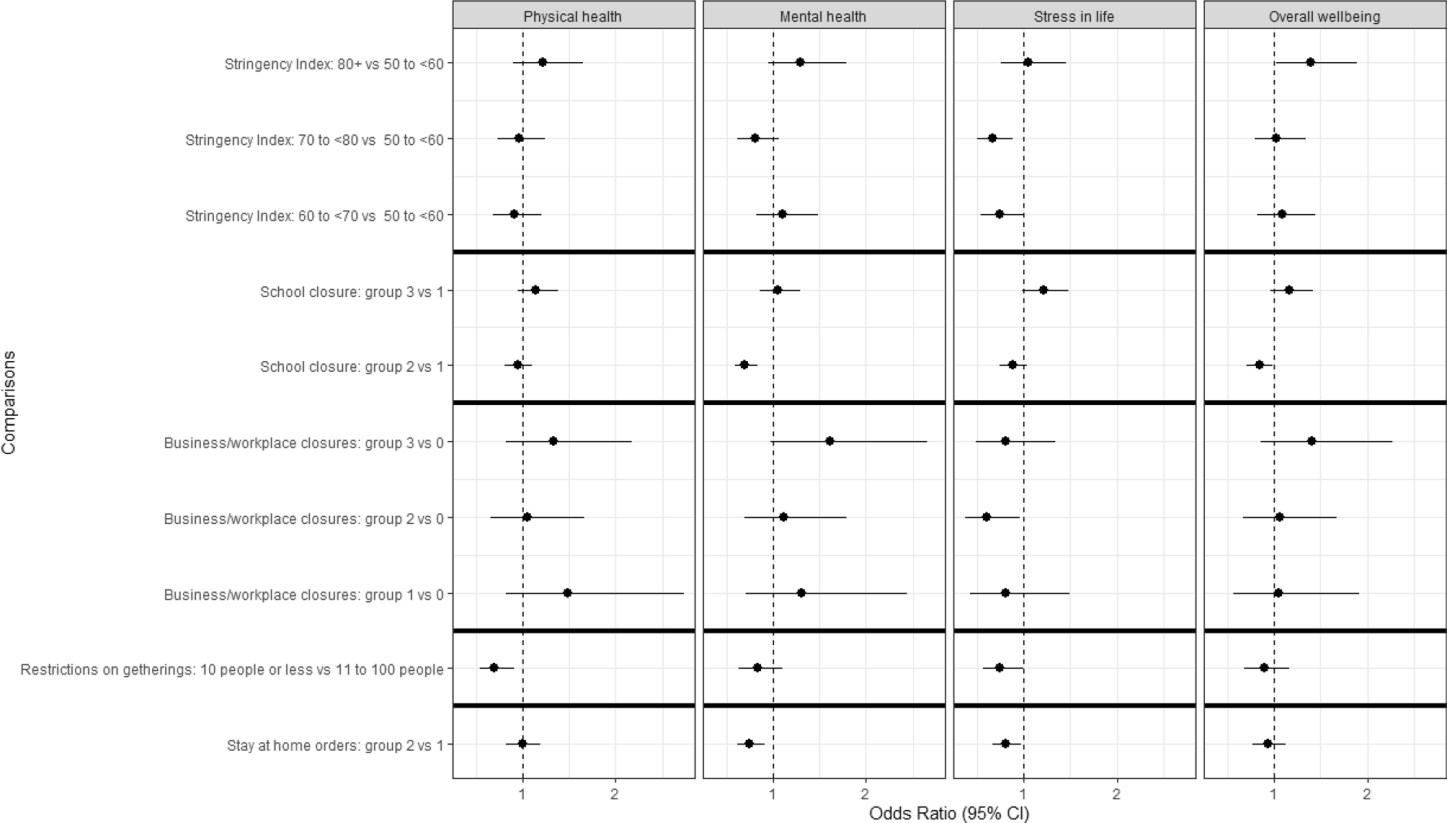
Adjusted odds ratios (95% confidence intervals) of feeling “somewhat/much worse” in each of the health related COVID-19 outcome vs “unchanged/improved” from government-mandated restrictions. Separate models for each government-mandated restriction were adjusted for age, gender, Indigenous status, geographical location, income, work status, general physical and mental health, anxiety/depression, 7-day rolling average rate of deaths due to COVID-19 and cases per 100,000. Solid black line indicates a separate model. Confidence intervals of odds ratios that include the value 1 are not considered statistically different from the reference group. The Hosmer–Lemeshow goodness of fit test was satisfied for all models (*p* > 0.05) except for mental health and school closures (*p* < 0.02), mental health and stay at home orders (*p* < 0.01) and mental health and stringency index (*p* < 0.01). School closures: group 3—all levels of school closed; group 2—some levels of school closed; group 1—schools recommended to close. Business/workplace closures: group 3—all non-essential businesses/workplaces closed; group 2—some non-essential businesses/workplaces closed; group 1—recommend to close non-essential businesses/workplaces; group 0—no closures. Restrictions on gatherings: group 4—10 people or less permitted to meet; group 3—11 to 100 people permitted to meet. Stay-at-home orders: group 2—leaving home permitted for essential trips; group 1—recommend not leave home.

A total of twenty multivariate logistic regression models were fit (i.e., for each of the four health-related COVID-19 outcome variables, by each of the main predictors of SI, school closure, business closures, restrictions on gatherings, stay at home orders). Full models are presented in Supplemental Tables S1–S5. Figure 6 presents the adjusted OR of those reporting “somewhat/much worse” in each of the health-related outcomes by SI group, school closure, business/workplace closures, restrictions on gatherings and stay-at-home orders (see Supplemental Tables S1–S5 for numerical OR values). Although there appeared to be an increase in the odds of reporting “somewhat/much worse” in each of the health-related outcomes when the SI group was 80+ , compared to when the SI was between 50 and less than 60, the increase only reached statistical significance for overall wellbeing. For example, the odds of feeling “somewhat/much worse” in one’s overall wellbeing was significantly higher by 39% among those who lived in an area with SI 80 + (OR = 1.39, 95% CI 1.02, 1.89, $\chi_{1}^{2}$ = 4.44, *p* = 0.035) compared to those living in areas with SI 50 to less than 60. Compared to people living in the areas with SI 50 to less than 60, significantly less people in areas with SI 60 to less than 70 and 70 to less than 80 reported that their stress in life worsened (SI between 60 and  < 70 vs reference group OR = 0.74, 95% CI 0.54, 1.00, $\chi_{1}^{2}$ = 3.88, *p* = 0.049; SI between 70 and < 80 vs reference group OR = 0.67, 95% CI 0.50, 0.88, $\chi_{1}^{2}$ = 8.27, *p* = 0.004).

A significantly lower odds of feeling “somewhat/much worse” for mental health and overall wellbeing was observed among those living in areas with a school closure rating of 2 (some levels closed) compared to a rating of 1 (recommended to close) (mental health OR = 0.70, 95% CI 0.59, 0.83, $\chi_{1}^{2}$ = 17.38, *p* < 0.001; overall wellbeing OR = 0.84, 95% CI 0.71, 0.99, $\chi_{1}^{2}$ = 4.59, *p* = 0.032). At the time of the survey, no respondents lived in areas with a school closure rating of 0.

In contrast, the odds of feeling “somewhat/much worse” stress in life was significantly lower among those with a business and workplace closure rating of 2 (some businesses/workplaces closed) compared to a rating of 0 (no closures) (OR = 0.60, 95% CI 0.37, 0.96, $\chi_{1}^{2}$ = 4.48, *p* = 0.034).

During the time of the survey, restrictions on gatherings were rated as 3 (11–100 people) or 4 (10 people or less), therefore a rating of 3 was considered the reference group. The odds of reporting “somewhat/much worse” for physical health (OR = 0.70, 95% CI 0.54, 0.91, $\chi_{1}^{2}$ = 6.86, p = 0.009) and stress in life (OR = 0.75, 95% CI 0.57, 1.00, $\chi_{1}^{2}$ = 3.95, *p* = 0.047) were significantly decreased among those living with greater restrictions on gatherings. Restrictions on gatherings were unrelated to the other health measures.

For the period of the survey, stay-at-home orders were only rated as 2 (required to stay-at-home with some exceptions) or 1 (recommend to stay-at-home), therefore those rated as 1 formed the reference group. A significant decrease in odds of feeling “somewhat/much worse” with respect to mental health (OR = 0.75, 95% CI 0.62, 0.91, $\chi_{1}^{2}$ = 8.51, *p* = 0.004) and stress in life (OR = 0.81, 95% CI 0.67, 0.98, $\chi_{1}^{2}$ = 4.83, *p* = 0.028) was observed among those with a rating of 2 compared to those with a rating of 1 with respect to stay-at-home orders. The odds of feeling “somewhat/much worse” with respect to physical health and overall wellbeing were similar between those living in an area with a stay-at-home order rated as 2 versus 1, but were not significant.

The impact of collapsing “somewhat worse” and “much worse” was assessed in a sensitivity analysis. The outcome of physical health, mental health, stress in life, and overall wellbeing were categorised as “much worse”, “somewhat worse” and “unchanged/improved” in a multinomial regression model. The reference group was defined as “unchanged/improved” in all models. When modelling the proportion of people who reported to be “much worse” or “somewhat worse” in a multinomial model the results were similar to the combined analysis, although the confidence intervals were much wider. By combining the results from the somewhat and much worse together the response and relationship between the COVID-19 health indicators and restrictions was more robust (i.e., smaller confidence intervals on the estimates) (Supplemental Tables S11 and S12). Furthermore, a separate multinomial model defining the outcomes as “somewhat/much worse”, “unchanged” and “somewhat/much improved” was also conducted in order to determine if there was an association between the “unchanged” and reference group “somewhat/much improved” of COVID-19 health outcomes and restrictions. No significant difference (*p* > 0.05) was observed, justifying the combination of the “unchanged” responses with the “somewhat/much improved” responses (data not shown).

## Discussion

This exploratory study provided some evidence that public health measures may have unintended impacts on self-reported health. Our findings are generally consistent with other publications that self-reported health was affected during the pandemic^1,7–9,21^, including recent observations from Statistics Canada^25^ reporting further decreases to self-rated mental health during the COVID-19 pandemic. In the univariate analyses, the strength of the overall SI was associated with a worsening of health, although there was a clear deviation from this general pattern when the SI was between 70 and < 80 (Fig. 4, univariate analysis). This pattern was less apparent when other variables (potential confounders) were adjusted for in the multivariate analyses. The multivariate analyses demonstrated that the association between SI and the outcome(s) was complex and impacted, at least partially by, the variables adjusted for. Similar complexities were observed when the individual public health measures were assessed. Potential explanations for the differences between the univariate and multivariate results are discussed below.

Given the unique circumstances that the COVID-19 pandemic presented, global citizens were heavily reliant on the choices that their respective governments did or did not put in place to protect against the virus. The current study included data that reflected the severity of restrictions placed on non-essential activities. As a composite measure of nine sub-components, the SI quantified the magnitude of government-imposed policies. It should be noted that while the SI can provide an indication of government restrictions, without analysis of the individual sub-components, and controlling for potential confounding variables, the SI alone cannot be used to evaluate the potential influence from any one particular policy (e.g., school closures). In general, the univariate analyses revealed an increase in the prevalence of worsening self-reported health status associated with differing levels of SI. There was a notable exception in the third highest SI category (i.e., between 70 and < 80) where the prevalence dropped in all four health outcomes. Several hypotheses may explain this drop, and have been observed in other studies, although not in direct comparison to the SI. The decrease may reflect a developed tolerance toward restrictions of this magnitude. Fancourt et al.^26^ observed a decline of self-reported anxiety and depressive symptoms 20 weeks after the initial enforced lockdown in England and postulated the decline may be due to adaptation of circumstances and gradual easing of stringent restrictions. The decrease observed in the current study may also be linked with increased trust that the government takes the threat of COVID-19 seriously as other studies have reported^27,28^. Other possible explanations for this drop in reporting of worsening self-reported health status might be: improved coping mechanisms; a feeling of collectively contributing toward “bending the pandemic curve” down; or the result of knowing that a ‘lockdown’ period is finite and near ending in some provinces. The rebound of reporting worsening self-reported health that occurred at the highest SI (80+) may suggest a developed despondence and inability to cope effectively with additional restrictions on human activity. These univariate analyses suggest that there may be a zone of greater tolerance for policy stringency (between 70 and < 80) where residents are perhaps relieved and comforted that governments are taking action to protect them, yet do not feel they have lost all autonomy and freedom. It is however unknown how this may change depending on the duration of the pandemic and at what point public health measures are introduced. Furthermore, the perceived impact of restrictions on one’s health may be influenced by seasonal factors (e.g., summer vs winter). These are areas that could be evaluated with future research.

Comparison of the unadjusted provincial results of reporting “somewhat/much worse” (Fig. 3) with respect to the four health outcomes present another pattern that may be worth further investigation: Upon visual inspection of the data, Alberta, Ontario, Saskatchewan, and Nova Scotia had the highest prevalence rates of reporting “somewhat/much worse” for health outcomes and all, but Ontario, experienced the greatest jump in SI value (refer to two modes of SI in Fig. 3) during the survey period compared to the previous year. Ontario, in contrast, remained at a similar modal SI value (between 90 and 100) for much of the pandemic leading into, and during the survey period. It is possible that sudden changes in stringency of policy, and prolonged periods of high stringency, may have greater impacts on the health of Canadians, especially stress in life and mental health. A divergence to these findings was Prince Edward Island where policy stringency had been consistently moderate throughout the pandemic period preceding and during the survey. Prince Edward Island experienced fewer periods of high policy stringency (70 + SI value) than other provinces and appears to have experienced the least deterioration of self-reported overall wellbeing.

It is worth noting that little is known about how the absence of strong public health interventions and resultant low SI values might affect self-reported health, either in Canada or globally. It is possible that an absence of such strong interventions, and the resultant cases and deaths due to COVID-19, may have resulted in significantly worse self-reported health (but further research would be warranted). Indeed, more stringent responses to the COVID-19 pandemic have been shown to be significantly associated with decreased deaths and incidence of COVID-19^29,30^, and a meta-analysis of research on the relationship between fear of COVID-19 and mental health revealed a strong correlation between fear of COVID-19 and anxiety, and a moderate to strong association between fear of COVID-19 and depression and stress^31^.

Investigation of four of the sub-components of the SI (chosen for evaluation based on their immediate potential to impact human interaction more directly than the other five SI sub-components) through multivariate models indicated that the strength of individual policies can positively or negatively impact the odds of reporting “somewhat/much worse” for all four categories of health, especially stress in life. Of note is that the odds of reporting worse stress in life and physical health appear to decrease in association with maximum restrictions on gatherings (level 4 vs 3). Likewise, reporting worse mental health and stress in life decreased at the maximum value for stay-at-home orders observed during the survey period (level 2 vs 1). Determining whether this finding is circumstantial due to timing of the survey with the third wave of the pandemic, constrained due to a lack of each policy indicator level for comparison, or whether there is a true compliance and social trust of stronger measures implemented for public safety, warrants further investigation.

Also of interest is the mid-range of individual policies that appear to be tolerated with decreased odds of reporting “somewhat/much worse” for mental health, stress in life and overall wellbeing. For school closures, the level associated with some levels being closed (an indicator value of 2) was associated with a decrease in reporting “somewhat/much worse” mental health and overall wellbeing when compared to the reference group (recommendations to close, value 1). It should be noted that ‘recommendations to close’ (value 1) in schools was associated with much variation in operational procedures across Canada and within provinces; some schools required cohorts to attend school at differing times, others did not, and some schools had very different hygiene/social distancing requirements. Similarly, for business/workplace closures, an indicator value of 2 was associated with unchanged/improved stress in life compared to the reference group—no restrictions (value of 0).

Both the univariate and multivariate findings could inform future research and policy decisions that aim to find a balance between limiting the adverse health impacts from a global pandemic and minimising the unintended consequences of over/under-restricting human interactions. Nevertheless, all models had residual uncertainty and much remains to be learned with respect to how public health measures during the pandemic may have impacted the selected health outcomes. While stringent anti-contagion policies have had large-scale positive impacts in preventing the spread of COVID-19 and subsequent illness and death in the current pandemic^32^, recognising which policies have had the strongest impacts on wellbeing may help policy-makers design and implement targeted mental health supports and social policies that enhance wellbeing in future pandemics—when even the most stringent policies may once again be necessary to save lives.

It is indisputable that answers to these unknowns may take several years to discover, where future research in this area will affirm/refute the variables considered and perhaps demonstrate the importance of those not considered in this study. It is reasonable to expect future studies will include a wider range of health outcomes, including those that are objectively measured; expanded population demographics, and different timelines.

## Limitations

The current study is limited due to the lack of a true ‘reference group’ for comparison of individual policy strength, and by low participant numbers for some of the response categories (much improved/somewhat improved/unchanged etc.) which required grouping. As CPENS was conducted for a discreet period, it did not allow for, nor provide data for a comparison of self-reported health measures during a period absent of any public protection measures/policies or following widespread COVID-19 vaccination availability. Similarly, the 10 provinces evaluated did not have a full range of OxCGRT restriction policy values to compare in the modelling. Nonetheless, the variation that we did observe was associated with some effects of note on self-reported health outcomes. Future studies should include an evaluation of the remaining five policy indicators of the OxCGRT Stringency Index (i.e., cancellation of public events, public transport closures, restrictions on internal movement, international travel controls and public health campaigns) to determine whether any of these sub-components may be central/significant to the precarious nature of total SI value and health outcomes.

It should be underscored that CPENS was not designed to provide a thorough evaluation of the impact that the pandemic may have had on Canadians’ health. Implementing validated psychometric tools for this purpose was beyond the scope of the survey. Furthermore, CPENS did not include questions about how COVID-19 may have affected relationships, family, or caring responsibilities. Although the impact that the pandemic has had on individuals under the age of 18 years^33^, and on those living in long-term care facilities^34^ has been disproportionate, these groups were not included in the sample. Furthermore, although CPENS evaluated the perceived impact that the pandemic had on self-reported health, it did not specifically probe how Canadians felt about the public health measures they were subject to. Studies that include both attitudes towards specific health measures and self-reported health could improve the understanding of the impact that such policies may have.

## Conclusion

In conclusion, the current study provides some evidence that Canadians’ self-reported mental health, stress in life and overall wellbeing may have been affected as a result of the pandemic, and the measures put in place to protect Canadians. Policies that are deemed either too stringent, or too lax, may have the potential to negatively affect mental health, stress in life, and overall wellbeing, while a middle ground may be better tolerated, with reduced effects on self-reported health. In general, public safety measures were associated with less impact on physical health when compared to the other measures evaluated. Future research and surveillance in this area may provide evidence as to what level of public safety measures are appropriate, with maximal tolerance and minimal negative effect on the health of Canadians.

### Supplementary Information

Supplementary Tables.

## Data Availability

The aggregated data tables are available as CSV files through Library and Archives Canada website: https://epe.lac-bac.gc.ca/100/200/301/pwgsc-tpsgc/por-ef/health/2021/133-20-e/index.html. The survey was originally entitled and administered as the “*Survey of Attitudes Towards Community Noise in Canada*”, changed to the *Canadian Perspectives on Environmental Noise Survey* in publications as the revised title more accurately captures the scope of the survey. All OxCGRT data is available via GitHub (https://github.com/OxCGRT/covid-policy-tracker) and is made available free to use for any purpose under a Creative Commons CC BY 4.0 license.
