# Supplementary material for: Variation in the stringency of COVID-19 public health measures on self-reported health, stress, and overall wellbeing in Canada
Source: Sci Rep. 2023 Aug 11;13:13094. doi: 10.1038/s41598-023-39004-w (PMC10421886; doi:10.1038/s41598-023-39004-w)
Supplement: Supplementary file 1 — Supplementary Tables. [file 41598_2023_39004_MOESM1_ESM.pdf]

Additional Supplemental Tables

Full logistic regression models for each COVID-19 related health outcome and COVID-19 restriction. Each column listed as "OR (95% CI)" represents a single model, with the odds ratio (OR) and 95% confidence interval for each parameter included in the model. All ORs are rounded to two decimal points.

Table S1: Adjusted odds ratios from logistic regression for the prevalence of "somewhat/much worse" versus "unchanged/improved" in each COVID-19 health related outcome and school closures

For each of the following, indicate how you have been personally affected by the COVID-19 pandemic (For each endpoint the probability of "Somewhat/much worse" is modelled, vs "unchanged/improved"). Odds ratio OR (95% confidence interval CI) is always compared to the reference category.

Variable	Categories	Physical health		Mental health		Stress in life		Overall wellbeing	
		**OR (95% CI)**	**χ^2_1, p-value [b]**	**OR (95% CI)**	**χ^2_1, p-value [b]**	**OR (95% CI)**	**χ^2_1, p-value [b]**	**OR (95% CI)**	**χ^2_1, p-value [b]**
N, HL p-value		5874, 0.22		5864, 0.02		5873, 0.45		5873, 0.76	
School Closures[a]	3=All levels of school closed	1.14 (0.95 - 1.38)	1.92, 0.166	1.05 (0.86 - 1.29)	0.23, 0.632	1.22 (0.99 - 1.49)	3.61, 0.057	1.17 (0.96 - 1.42)	2.45, 0.117
	2=Some levels of school closed	0.95 (0.81 - 1.11)	0.37, 0.543	0.70 (0.59 - 0.83)	17.38, <0.001	0.88 (0.74 - 1.04)	2.38, 0.123	0.84 (0.71 - 0.99)	4.59, 0.032
	1=Schools recommended to close	Reference		Reference		Reference		Reference	
	0=No restrictions	No data		No data		No data		No data	
Age group	18 to 34	1.38 (1.16 - 1.64)	13.63, <0.001	1.74 (1.45 - 2.09)	35.45, <0.001	1.43 (1.19 - 1.73)	14.49, <0.001	1.27 (1.07 - 1.51)	7.14, 0.008
	35 to 54	1.25 (1.07 - 1.47)	7.75, 0.005	1.38 (1.17 - 1.62)	14.82, <0.001	1.22 (1.03 - 1.44)	5.35, 0.021	1.18 (1.00 - 1.38)	3.96, 0.047
	55+	Reference		Reference		Reference		Reference	
Gender	Other/Prefer not to say	1.20 (0.70 - 2.08)	0.44, 0.506	2.27 (1.01 - 5.08)	3.97, 0.046	0.89 (0.47 - 1.68)	0.13, 0.719	0.92 (0.51 - 1.67)	0.07, 0.785
	Female	0.87 (0.78 - 0.98)	5.67, 0.017	1.14 (1.02 - 1.29)	4.97, 0.026	1.33 (1.18 - 1.5)	21.79, <0.001	1.03 (0.92 - 1.16)	0.28, 0.595
	Male	Reference		Reference		Reference		Reference	
Indigenous status	Indigenous	1.18 (0.92 - 1.52)	1.76, 0.184	0.99 (0.76 - 1.3)	0.00, 0.958	1.04 (0.79 - 1.38)	0.08, 0.781	1.02 (0.78 - 1.32)	0.01, 0.903
	Non-Indigenous	Reference		Reference		Reference		Reference	
Geographical area	Urban	1.25 (1.06 - 1.46)	7.12, 0.008	1.10 (0.93 - 1.31)	1.25, 0.263	1.12 (0.94 - 1.32)	1.58, 0.209	1.18 (1.01 - 1.40)	4.09, 0.043

	Suburban	1.21 (1.04 - 1.41)	5.95, 0.015	1.11 (0.94 - 1.3)	1.50, 0.220	1.17 (0.99 - 1.37)	3.57, 0.059	1.16 (0.99 - 1.35)	3.34, 0.068
	Rural/remote	Reference		Reference		Reference		Reference	
Income	$150K or more	0.98 (0.81 - 1.19)	0.05, 0.822	1.28 (1.04 - 1.57)	5.31, 0.021	1.38 (1.11 - 1.70)	8.76, 0.003	1.28 (1.05 - 1.57)	5.96, 0.015
	$80K - <$150K	1.10 (0.93 - 1.29)	1.27, 0.260	1.19 (1.00 - 1.43)	3.79, 0.052	1.21 (1.01 - 1.44)	4.23, 0.040	1.14 (0.96 - 1.35)	2.12, 0.145
	$40K - <$80K	1.03 (0.87 - 1.21)	0.11, 0.742	1.05 (0.88 - 1.26)	0.30, 0.584	1.07 (0.89 - 1.28)	0.50, 0.478	0.98 (0.83 - 1.17)	0.05, 0.829
	less than $40K	Reference		Reference		Reference		Reference	
Work/attend school outside home	Yes	0.87 (0.70 - 1.07)	1.84, 0.175	1.08 (0.86 - 1.35)	0.42, 0.518	1.06 (0.84 - 1.34)	0.27, 0.603	1.01 (0.81 - 1.26)	0.01, 0.920
	No	Reference		Reference		Reference		Reference	
Work/attend school inside home	Yes	1.04 (0.85 - 1.28)	0.17, 0.682	0.98 (0.79 - 1.23)	0.02, 0.893	1.03 (0.82 - 1.29)	0.05, 0.820	0.97 (0.78 - 1.20)	0.08, 0.781
	No	Reference		Reference		Reference		Reference	
Retired	Yes	0.85 (0.66 - 1.10)	1.47, 0.226	0.75 (0.57 - 0.99)	4.23, 0.040	0.74 (0.56 - 0.97)	4.68, 0.031	0.76 (0.58 - 0.98)	4.41, 0.036
	No	Reference		Reference		Reference		Reference	
Unemployed	Yes	1.04 (0.80 - 1.36)	0.09, 0.758	1.08 (0.80 - 1.45)	0.27, 0.606	1.19 (0.88 - 1.60)	1.21, 0.270	1.04 (0.79 - 1.38)	0.09, 0.765
	No	Reference		Reference		Reference		Reference	
On paid leave (sick leave, maternity, disability)	Yes	0.61 (0.42 - 0.90)	6.16, 0.013	1.27 (0.81 - 1.98)	1.07, 0.301	1.01 (0.65 - 1.58)	0.00, 0.952	1.23 (0.81 - 1.88)	0.93, 0.336
	No	Reference		Reference		Reference		Reference	
Other	Yes	1.10 (0.78 - 1.55)	0.28, 0.600	1.23 (0.85 - 1.77)	1.18, 0.277	1.05 (0.73 - 1.53)	0.08, 0.780	0.90 (0.63 - 1.29)	0.31, 0.576
	No	Reference		Reference		Reference		Reference	
Rated physical health in general, for one's age	Fair/poor	1.77 (1.52 - 2.07)	52.83, <0.001	0.94 (0.79 - 1.12)	0.55, 0.458	1.29 (1.07 - 1.54)	7.44, 0.006	1.32 (1.12 - 1.56)	10.69, 0.001
	Excellent/very good/good	Reference		Reference		Reference		Reference	
Rated mental health in general, for one's age	Fair/poor	1.50 (1.29 - 1.75)	26.28, <0.001	3.47 (2.83 - 4.26)	143.33, <0.001	2.26 (1.84 - 2.76)	62.77, <0.001	2.62 (2.19 - 3.14)	111.69, <0.001
	Excellent/very good/good	Reference		Reference		Reference		Reference	
Anxiety or depression	Diagnosed	1.57 (1.34 - 1.83)	32.17, <0.001	1.99 (1.67 - 2.35)	62.19, <0.001	1.59 (1.33 - 1.89)	27.23, <0.001	1.95 (1.66 - 2.30)	65.24, <0.001
	Suffer	1.56 (1.35 - 1.80)	35.67, <0.001	2.88 (2.44 - 3.41)	153.72, <0.001	2.50 (2.10 - 2.97)	105.00, <0.001	2.43 (2.08 - 2.83)	126.36, <0.001
	Does not apply	Reference		Reference		Reference		Reference	

Rate of deaths during the previous 7 days	0.98 (0.80 - 1.21)	0.04, 0.845	0.84 (0.68 - 1.05)	2.27, 0.132	0.67 (0.53 - 0.84)	12.39, <0.001	0.94 (0.76 - 1.16)	0.36, 0.546
Rate of cases during the previous 7 days [c]	1.24 (1.13 - 1.37)	20.25, <0.001	1.32 (1.19 – 1.46)	28.52, <0.001	1.47 (1.32 – 1.64)	51.34, <0.001	1.20 (1.09 – 1.32)	13.52, <0.001

N number of complete observations used in the model; HL Hosmer-Lemeshow goodness of fit test, if p>0.05 then the model fits the data well; [a] people are assigned the OxCGRT value for school closures that corresponds to the date they filled out the survey; [b] Wald Chi-square test from logistic regression with 1 degree of freedom comparing each level to the reference level within a variable, p-value based on Bonferroni correction. [c] OR is given for every 100 new cases reported. In instances where the confidence interval of the OR includes 1.00 yet is significant p<0.05, this is due to rounding error.

Table S2: Adjusted odds ratios from logistic regression for the prevalence of "somewhat/much worse" versus "unchanged/improved" in each COVID-19 health related outcome and business/workplace closure

For each of the following, indicate how you have been personally affected by the COVID-19 pandemic (For each endpoint the probability of "Somewhat/much worse" is modelled, vs "unchanged/improved"). Odds ratio OR (95% confidence interval CI) is always compared to the reference category.

Variable	Categories	**Physical health**		**Mental health**		**Stress in life**		**Overall wellbeing**	
		**OR (95% CI)**	**χ^2_1, p-value [b]**	**OR (95% CI)**	**χ^2_1, p-value [b]**	**OR (95% CI)**	**χ^2_1, p-value [b]**	**OR (95% CI)**	**χ^2_1, p-value [b]**
N, HL p-value		5874, 0.07		5864, 0.47		5873, 0.15		5873, 0.23	
Business/ workplace closures [a]	3=All non-essential businesses/workplaces closed	1.33 (0.82 - 2.17)	1.32, 0.250	1.61 (0.98 - 2.66)	3.49, 0.062	0.81 (0.49 - 1.35)	0.65, 0.421	1.40 (0.86 - 2.27)	1.82, 0.178
	2=Some non-essential businesses/workplaces closed	1.05 (0.66 - 1.67)	0.04, 0.843	1.12 (0.70 - 1.79)	0.22, 0.643	0.60 (0.37 - 0.96)	4.48, 0.034	1.06 (0.67 - 1.67)	0.06, 0.809
	1=Recommend to close non-essential businesses/workplaces	1.49 (0.82 - 2.73)	1.72, 0.190	1.31 (0.71 - 2.44)	0.75, 0.385	0.81 (0.43 - 1.50)	0.45, 0.501	1.05 (0.57 - 1.91)	0.02, 0.883
	0=No closures	Reference		Reference		Reference		Reference	
Age group	18 to 34	1.38 (1.16 - 1.64)	13.58, <0.001	1.72 (1.43 - 2.06)	34.25, <0.001	1.42 (1.18 - 1.71)	13.87, <0.001	1.27 (1.06 - 1.51)	6.95, 0.008
	35 to 54	1.25 (1.07 - 1.47)	7.78, 0.005	1.36 (1.15 - 1.60)	13.44, <0.001	1.21 (1.03 - 1.43)	5.11, 0.024	1.17 (0.99 - 1.37)	3.58, 0.058
	55+	Reference		Reference		Reference		Reference	
Gender	Other/Prefer not to say	1.20 (0.69 - 2.07)	0.43, 0.513	2.27 (1.02 - 5.07)	4.02, 0.045	0.88 (0.47 - 1.65)	0.16, 0.689	0.92 (0.51 - 1.67)	0.07, 0.791
	Female	0.88 (0.78 - 0.98)	5.48, 0.019	1.14 (1.02 - 1.29)	4.93, 0.026	1.33 (1.18 - 1.50)	21.61, <0.001	1.03 (0.92 - 1.16)	0.29, 0.593
	Male	Reference		Reference		Reference		Reference	
Indigenous status	Indigenous	1.18 (0.92 - 1.52)	1.74, 0.187	1.00 (0.76 - 1.31)	0.00, 0.980	1.04 (0.79 - 1.37)	0.07, 0.785	1.01 (0.78 - 1.32)	0.01, 0.919
	Non-Indigenous	Reference		Reference		Reference		Reference	
Geographical area	Urban	1.25 (1.06 - 1.46)	7.07, 0.008	1.10 (0.93 - 1.30)	1.17, 0.279	1.11 (0.94 - 1.32)	1.45, 0.229	1.18 (1.00 - 1.39)	3.92, 0.048
	Suburban	1.22 (1.04 - 1.42)	6.29, 0.012	1.12 (0.95 - 1.31)	1.90, 0.168	1.18 (1 - 1.38)	3.93, 0.047	1.16 (0.99 - 1.36)	3.59, 0.058
	Rural/remote	Reference		Reference		Reference		Reference	
Income	$150K or more	0.97 (0.8 - 1.18)	0.09, 0.768	1.28 (1.04 - 1.58)	5.50, 0.019	1.36 (1.10 - 1.68)	8.08, 0.004	1.29 (1.05 - 1.57)	6.14, 0.013

	$80K - <$150K	1.09 (0.93 - 1.29)	1.10, 0.295	1.19 (0.99 - 1.42)	3.53, 0.060	1.19 (1.00 - 1.43)	3.71, 0.054	1.13 (0.96 - 1.35)	2.11, 0.146
	$40K - <$80K	1.03 (0.87 - 1.21)	0.10, 0.755	1.05 (0.88 - 1.26)	0.28, 0.599	1.06 (0.88 - 1.27)	0.39, 0.530	0.98 (0.83 - 1.17)	0.04, 0.833
	less than $40K	Reference		Reference		Reference		Reference	
Work/attend school outside home	Yes	0.87 (0.71 - 1.07)	1.72, 0.190	1.07 (0.85 - 1.34)	0.34, 0.561	1.06 (0.84 - 1.33)	0.25, 0.617	1.00 (0.81 - 1.25)	0.00, 0.969
	No	Reference		Reference		Reference		Reference	
Work/attend school inside home	Yes	1.05 (0.85 - 1.29)	0.21, 0.649	0.97 (0.77 - 1.21)	0.09, 0.758	1.02 (0.82 - 1.29)	0.05, 0.832	0.96 (0.78 - 1.19)	0.14, 0.707
	No	Reference		Reference		Reference		Reference	
Retired	Yes	0.85 (0.66 - 1.10)	1.47, 0.225	0.74 (0.57 - 0.97)	4.77, 0.029	0.73 (0.56 - 0.96)	4.97, 0.026	0.75 (0.57 - 0.97)	4.79, 0.029
	No	Reference		Reference		Reference		Reference	
Unemployed	Yes	1.05 (0.80 - 1.37)	0.12, 0.729	1.09 (0.81 - 1.46)	0.29, 0.587	1.19 (0.88 - 1.61)	1.22, 0.268	1.05 (0.79 - 1.39)	0.10, 0.749
	No	Reference		Reference		Reference		Reference	
On paid leave (sick leave, maternity, disability)	Yes	0.62 (0.42 - 0.91)	5.82, 0.016	1.31 (0.83 - 2.05)	1.36, 0.244	1.03 (0.66 - 1.60)	0.01, 0.911	1.25 (0.82 - 1.91)	1.06, 0.304
	No	Reference		Reference		Reference		Reference	
Other	Yes	1.10 (0.78 - 1.56)	0.30, 0.584	1.21 (0.84 - 1.75)	1.01, 0.315	1.05 (0.72 - 1.52)	0.06, 0.801	0.89 (0.63 - 1.27)	0.39, 0.532
	No	Reference		Reference		Reference		Reference	
Rated physical health in general, for one's age	Fair/poor	1.77 (1.52 - 2.07)	52.70, <0.001	0.93 (0.78 - 1.11)	0.58, 0.445	1.28 (1.07 - 1.53)	7.22, 0.007	1.32 (1.12 - 1.56)	10.82, 0.001
	Excellent/very good/good	Reference		Reference		Reference		Reference	
Rated mental health in general, for one's age	Fair/poor	1.50 (1.28 - 1.75)	25.88, <0.001	3.45 (2.82 - 4.23)	142.18, <0.001	2.25 (1.84 - 2.75)	62.41, <0.001	2.62 (2.19 - 3.13)	111.11, <0.001
	Excellent/very good/good	Reference		Reference		Reference		Reference	
Anxiety or depression	Diagnosed	1.57 (1.34 - 1.83)	32.07, <0.001	2.00 (1.68 - 2.37)	63.34, <0.001	1.59 (1.34 - 1.90)	27.69, <0.001	1.96 (1.67 - 2.30)	66.03, <0.001
	Suffer	1.55 (1.34 - 1.80)	34.96, <0.001	2.89 (2.45 - 3.42)	154.98, <0.001	2.49 (2.09 - 2.97)	104.51, <0.001	2.43 (2.08 - 2.84)	126.92, <0.001
	Does not apply	Reference		Reference		Reference		Reference	
Rate of deaths during the previous 7 days		0.92 (0.74 - 1.13)	0.62, 0.430	0.74 (0.59 - 0.92)	7.23, 0.007	0.66 (0.52 - 0.82)	13.39, <0.001	0.89 (0.72 - 1.11)	1.05, 0.304
Rate of cases during the previous 7 days[c]		1.28 (1.16 – 1.41)	23.52, <0.001	1.36 (1.22 - 1.51)	31.50, <0.001	1.56 (1.39 – 1.74)	61.77, <0.001	1.22 (1.10 - 1.35)	14.46, <0.001

N number of complete observations used in the model; HL Hosmer-Lemeshow goodness of fit test, if p>0.05 then the model fits the data well; [a] people are assigned the OxCGRT value for business/workplace closures that corresponds to the date they filled out the survey; [b] Wald Chi-square test from logistic regression with 1 degree of freedom comparing each level to the reference level within a variable, p-value based on Bonferroni correction; [c] OR is given for every 100 new cases reported. In instances where the confidence interval of the OR includes 1.00 yet is significant p<0.05, this is due to rounding error.

Table S3: Adjusted odds ratios from logistic regression for the prevalence of "somewhat/much worse" versus "unchanged/improved" in each COVID-19 health related outcome and restrictions on gatherings

For each of the following, indicate how you have been personally affected by the COVID-19 pandemic (For each endpoint the probability of "Somewhat/much worse" is modelled, vs "unchanged/improved"). Odds ratio OR (95% confidence interval CI) is always compared to the reference category.

Variable	Categories	**Physical health**		**Mental health**		**Stress in life**		**Overall wellbeing**	
		**OR (95% CI)**	**χ^2_1, p-value [b]**	**OR (95% CI)**	**χ^2_1, p-value [b]**	**OR (95% CI)**	**χ^2_1, p-value [b]**	**OR (95% CI)**	**χ^2_1, p-value [b]**
N, HL p-value		5874, 0.51		5864, 0.06		5873, 0.36		5873, 0.21	
Restrictions on gatherings [a]	4 = ≤10 people	0.70 (0.54 - 0.91)	6.86, 0.009	0.83 (0.63 - 1.10)	1.61, 0.205	0.75 (0.57 – 1.00)	3.95, 0.047	0.90 (0.68 - 1.17)	0.64, 0.425
	3 = 11-100 people	Reference		Reference		Reference		Reference	
	2 = 101-1000 people	No data		No data		No data		No data	
	1 = >1000 people	No data		No data		No data		No data	
	0 = no restrictions	No data		No data		No data		No data	
Age group	18 to 34	1.38 (1.16 - 1.64)	13.52, <0.001	1.72 (1.43 - 2.06)	34.06, <0.001	1.43 (1.19 - 1.72)	14.21, <0.001	1.26 (1.06 - 1.51)	6.90, 0.009
	35 to 54	1.24 (1.06 - 1.46)	7.36, 0.007	1.35 (1.14 - 1.58)	12.83, <0.001	1.20 (1.02 - 1.42)	4.80, 0.028	1.16 (0.99 - 1.36)	3.36, 0.067
	55+	Reference		Reference		Reference		Reference	
Gender	Other/Prefer not to say	1.20 (0.69 - 2.07)	0.43, 0.513	2.28 (1.02 - 5.09)	4.08, 0.043	0.89 (0.47 - 1.67)	0.14, 0.712	0.93 (0.51 - 1.68)	0.06, 0.808
	Female	0.87 (0.78 - 0.97)	6.10, 0.013	1.13 (1.00 - 1.27)	4.07, 0.044	1.32 (1.17 - 1.48)	20.42, <0.001	1.02 (0.91 - 1.14)	0.13, 0.713
	Male	Reference		Reference		Reference		Reference	
Indigenous status	Indigenous	1.16 (0.90 - 1.49)	1.37, 0.241	0.98 (0.74 - 1.28)	0.03, 0.857	1.01 (0.77 - 1.34)	0.01, 0.930	1.00 (0.77 - 1.29)	0.00, 0.985
	Non-Indigenous	Reference		Reference		Reference		Reference	
Geographical area	Urban	1.23 (1.05 - 1.45)	6.47, 0.011	1.09 (0.92 - 1.29)	1.03, 0.310	1.10 (0.93 - 1.31)	1.30, 0.254	1.18 (1.00 - 1.38)	3.75, 0.053
	Suburban	1.21 (1.04 - 1.41)	5.98, 0.014	1.11 (0.95 - 1.30)	1.72, 0.189	1.17 (1.00 - 1.37)	3.69, 0.055	1.16 (0.99 - 1.35)	3.44, 0.064
	Rural/remote	Reference		Reference		Reference		Reference	
Income	$150K or more	0.97 (0.80 - 1.18)	0.07, 0.791	1.29 (1.05 - 1.59)	5.91, 0.015	1.38 (1.12 - 1.71)	9.01, 0.003	1.30 (1.06 - 1.58)	6.46, 0.011

	$80K - <$150K	1.10 (0.93 - 1.29)	1.26, 0.261	1.20 (1.01 - 1.44)	4.15, 0.042	1.21 (1.02 - 1.45)	4.53, 0.033	1.15 (0.97 - 1.36)	2.48, 0.116
	$40K - <$80K	1.02 (0.87 - 1.21)	0.08, 0.777	1.05 (0.88 - 1.26)	0.31, 0.579	1.07 (0.89 - 1.28)	0.48, 0.489	0.98 (0.83 - 1.17)	0.04, 0.847
	less than $40K	Reference		Reference		Reference		Reference	
Work/attend school outside home	Yes	0.86 (0.70 - 1.06)	1.96, 0.161	1.05 (0.84 - 1.32)	0.19, 0.666	1.05 (0.83 - 1.32)	0.16, 0.690	0.99 (0.80 - 1.23)	0.00, 0.954
	No	Reference		Reference		Reference		Reference	
Work/attend school inside home	Yes	1.05 (0.86 - 1.29)	0.23, 0.633	0.96 (0.77 - 1.20)	0.11, 0.740	1.02 (0.82 - 1.28)	0.04, 0.839	0.96 (0.77 - 1.19)	0.15, 0.701
	No	Reference		Reference		Reference		Reference	
Retired	Yes	0.85 (0.65 - 1.09)	1.64, 0.201	0.73 (0.56 - 0.95)	5.27, 0.022	0.73 (0.55 - 0.95)	5.31, 0.021	0.74 (0.57 - 0.96)	5.16, 0.023
	No	Reference		Reference		Reference		Reference	
Unemployed	Yes	1.05 (0.80 - 1.37)	0.12, 0.730	1.08 (0.80 - 1.45)	0.25, 0.618	1.19 (0.88 - 1.60)	1.23, 0.268	1.04 (0.79 - 1.38)	0.09, 0.761
	No	Reference		Reference		Reference		Reference	
On paid leave (sick leave, maternity, disability)	Yes	0.62 (0.42 - 0.91)	5.91, 0.015	1.28 (0.82 - 2.01)	1.19, 0.276	1.02 (0.65 - 1.60)	0.01, 0.926	1.24 (0.81 - 1.89)	0.97, 0.324
	No	Reference		Reference		Reference		Reference	
Other	Yes	1.08 (0.77 - 1.53)	0.20, 0.658	1.19 (0.82 - 1.72)	0.85, 0.356	1.03 (0.71 - 1.50)	0.03, 0.861	0.88 (0.62 - 1.26)	0.47, 0.491
	No	Reference		Reference		Reference		Reference	
Rated physical health in general, for one's age	Fair/poor	1.77 (1.51 - 2.06)	52.31, <0.001	0.94 (0.79 - 1.12)	0.54, 0.462	1.29 (1.07 - 1.54)	7.52, 0.006	1.32 (1.12 - 1.57)	10.88, <0.001
	Excellent/very good/good	Reference		Reference		Reference		Reference	
Rated mental health in general, for one's age	Fair/poor	1.51 (1.29 - 1.77)	27.10, <0.001	3.50 (2.85 - 4.29)	145.43, <0.001	2.28 (1.87 - 2.79)	64.55, <0.001	2.64 (2.21 - 3.16)	113.78, <0.001
	Excellent/very good/good	Reference		Reference		Reference		Reference	
Anxiety or depression	Diagnosed	1.57 (1.35 - 1.84)	32.78, <0.001	2.00 (1.69 - 2.38)	64.40, <0.001	1.60 (1.34 - 1.90)	28.19, <0.001	1.97 (1.67 - 2.31)	66.95, <0.001
	Suffer	1.56 (1.35 - 1.80)	35.68, <0.001	2.91 (2.46 - 3.44)	157.45, <0.001	2.5.0 (2.10 - 2.98)	106.08, <0.001	2.44 (2.09 - 2.85)	128.72, <0.001
	Does not apply	Reference		Reference		Reference		Reference	
Rate of deaths during the previous 7 days		1.16 (0.98 - 1.37)	2.83, 0.092	1.02 (0.85 - 1.22)	0.04, 0.840	0.83 (0.69 - 1.00)	3.74, 0.053	1.15 (0.97 - 1.37)	2.47, 0.116
Rate of cases during the previous 7 days [c]		1.30 (1.17 - 1.43)	25.83, <0.001	1.36 (1.22 – 1.51)	30.91, <0.001	1.54 (1.37 – 1.72)	56.33, <0.001	1.22 (1.10 – 1.35)	14.20, <0.001

N number of complete observations used in the model; HL Hosmer-Lemeshow goodness of fit test, if $p>0.05$ then the model fits the data well; [a] people are assigned the OxCGRT value for restrictions on gatherings that corresponds to the date they filled out the survey; [b] Wald Chi-square test from logistic regression with 1 degree of freedom comparing each level to the reference level within a variable, p-value based on Bonferroni correction; [c] OR is given for every 100 new cases reported. In instances where the confidence interval of the OR includes 1.00 yet is significant $p<0.05$, this is due to rounding error.

Table S4: Adjusted odds ratios from logistic regression for the prevalence of "somewhat/much worse" versus "unchanged/improved" in each COVID-19 health related outcome and stay at home orders

For each of the following, indicate how you have been personally affected by the COVID-19 pandemic (For each endpoint the probability of "Somewhat/much worse" is modelled, vs "unchanged/improved"). Odds ratio OR (95% confidence interval CI) is always compared to the reference category.

Variable	Categories	**Physical health**		**Mental health**		**Stress in life**		**Overall wellbeing**	
		**OR (95% CI)**	**χ^2_1, p-value [b]**	**OR (95% CI)**	**χ^2_1, p-value [b]**	**OR (95% CI)**	**χ^2_1, p-value [b]**	**OR (95% CI)**	**χ^2_1, p-value [b]**
N, HL p-value		5874, 0.49		5864, 0.003		5873, 0.53		5873, 0.33	
Stay at home orders [a]	3= Leaving home permitted for minimal exceptions	No data		No data		No data		No data	
	2=Leaving home permitted for essential trips	1.00 (0.83 - 1.20)	0.00, 0.996	0.75 (0.62 - 0.91)	8.51, 0.004	0.81 (0.67 - 0.98)	4.83, 0.028	0.93 (0.77 - 1.12)	0.59, 0.441
	1=Recommend not leave home	Reference		Reference		Reference		Reference	
	0=No restrictions	No data		No data		No data		No data	
Age group	18 to 34	1.38 (1.16 - 1.64)	13.65, <0.001	1.72 (1.44 - 2.06)	34.52, <0.001	1.43 (1.19 - 1.72)	14.47, <0.001	1.27 (1.06 - 1.51)	6.97, 0.008
	35 to 54	1.25 (1.06 - 1.46)	7.42, 0.006	1.36 (1.16 - 1.60)	13.72, <0.001	1.21 (1.03 - 1.43)	5.18, 0.023	1.16 (0.99 - 1.36)	3.47, 0.062
	55+	Reference		Reference		Reference		Reference	
Gender	Other/Prefer not to say	1.21 (0.70 - 2.09)	0.46, 0.496	2.29 (1.02 - 5.10)	4.08, 0.043	0.89 (0.48 - 1.67)	0.13, 0.722	0.93 (0.51 - 1.68)	0.06, 0.811
	Female	0.87 (0.78 - 0.97)	6.07, 0.014	1.13 (1.00 - 1.27)	4.00, 0.045	1.32 (1.17 - 1.48)	20.35, <0.001	1.02 (0.91 - 1.14)	0.13, 0.715
	Male	Reference		Reference		Reference		Reference	
Indigenous status	Indigenous	1.17 (0.91 - 1.50)	1.54, 0.215	0.95 (0.72 - 1.25)	0.13, 0.720	1.00 (0.75 - 1.32)	0.00, 0.983	0.99 (0.76 - 1.29)	0.00, 0.958
	Non-Indigenous	Reference		Reference		Reference		Reference	
Geographical area	Urban	1.24 (1.06 - 1.46)	6.93, 0.008	1.09 (0.92 - 1.29)	1.03, 0.309	1.11 (0.93 - 1.31)	1.40, 0.237	1.18 (1.00 - 1.39)	3.82, 0.051
	Suburban	1.21 (1.04 - 1.41)	5.92, 0.015	1.11 (0.95 - 1.30)	1.63, 0.202	1.17 (0.99 - 1.37)	3.57, 0.059	1.16 (0.99 - 1.35)	3.40, 0.065
	Rural/remote	Reference		Reference		Reference		Reference	
Income	$150K or more	0.98 (0.81 - 1.20)	0.02, 0.874	1.28 (1.04 - 1.58)	5.55, 0.018	1.38 (1.12 - 1.70)	8.97, 0.003	1.30 (1.06 - 1.58)	6.46, 0.011

	$80K - <$150K	1.11 (0.94 - 1.30)	1.50, 0.221	1.20 (1.01 - 1.44)	4.13, 0.042	1.22 (1.02 - 1.45)	4.70, 0.030	1.15 (0.97 - 1.36)	2.54, 0.111
	$40K - <$80K	1.03 (0.87 - 1.22)	0.12, 0.725	1.05 (0.88 - 1.26)	0.32, 0.574	1.07 (0.89 - 1.28)	0.54, 0.463	0.98 (0.83 - 1.17)	0.03, 0.859
	less than $40K	Reference		Reference		Reference		Reference	
Work/attend school outside home	Yes	0.86 (0.70 - 1.06)	2.10, 0.147	1.05 (0.84 - 1.32)	0.21, 0.651	1.05 (0.83 - 1.32)	0.16, 0.690	0.99 (0.80 - 1.23)	0.00, 0.951
	No	Reference		Reference		Reference		Reference	
Work/attend school inside home	Yes	1.04 (0.85 - 1.27)	0.13, 0.718	0.97 (0.78 - 1.22)	0.06, 0.810	1.03 (0.82 - 1.29)	0.05, 0.819	0.96 (0.78 - 1.19)	0.15, 0.703
	No	Reference		Reference		Reference		Reference	
Retired	Yes	0.84 (0.65 - 1.09)	1.67, 0.196	0.74 (0.56 - 0.96)	5.02, 0.025	0.73 (0.56 - 0.96)	5.17, 0.023	0.74 (0.57 - 0.96)	5.10, 0.024
	No	Reference		Reference		Reference		Reference	
Unemployed	Yes	1.04 (0.80 - 1.36)	0.10, 0.754	1.08 (0.81 - 1.45)	0.28, 0.599	1.19 (0.88 - 1.61)	1.26, 0.262	1.05 (0.79 - 1.38)	0.10, 0.757
	No	Reference		Reference		Reference		Reference	
On paid leave (sick leave, maternity, disability)	Yes	0.62 (0.42 - 0.91)	6.09, 0.014	1.26 (0.81 - 1.97)	1.03, 0.311	1.01 (0.64 - 1.57)	0.00, 0.982	1.23 (0.81 - 1.88)	0.92, 0.337
	No	Reference		Reference		Reference		Reference	
Other	Yes	1.08 (0.77 - 1.53)	0.21, 0.647	1.20 (0.83 - 1.73)	0.93, 0.335	1.04 (0.72 - 1.51)	0.04, 0.833	0.88 (0.62 - 1.26)	0.46, 0.500
	No	Reference		Reference		Reference		Reference	
Rated physical health in general, for one's age	Fair/poor	1.77 (1.52 - 2.07)	53.19, <0.001	0.94 (0.79 - 1.12)	0.53, 0.465	1.29 (1.08 - 1.55)	7.70, 0.006	1.33 (1.12 - 1.57)	10.98, <0.001
	Excellent/very good/good	Reference		Reference		Reference		Reference	
Rated mental health in general, for one's age	Fair/poor	1.51 (1.29 - 1.76)	27.09, <0.001	3.51 (2.86 - 4.30)	146.15, <0.001	2.29 (1.87 - 2.80)	64.84, <0.001	2.65 (2.21 - 3.16)	113.93, <0.001
	Excellent/very good/good	Reference		Reference		Reference		Reference	
Anxiety or depression	Diagnosed	1.57 (1.35 - 1.84)	32.80, <0.001	2.00 (1.69 - 2.37)	63.66, <0.001	1.59 (1.34 - 1.90)	27.77, <0.001	1.97 (1.67 - 2.31)	66.70, <0.001
	Suffer	1.56 (1.35 - 1.81)	36.38, <0.001	2.90 (2.45 - 3.43)	156.25, <0.001	2.50 (2.10 - 2.98)	105.63, <0.001	2.44 (2.09 - 2.85)	128.54, <0.001
	Does not apply	Reference		Reference		Reference		Reference	
Rate of deaths during the previous 7 days		1.12 (0.86 - 1.46)	0.71, 0.398	1.38 (1.04 - 1.83)	5.10, 0.024	1.04 (0.78 - 1.38)	0.06, 0.807	1.24 (0.94 - 1.62)	2.36, 0.125
Rate of cases during the previous 7 days [c]		1.24 (1.11 – 1.39)	14.45, <0.001	1.21 (1.07 – 1.36)	9.23, 0.002	1.38 (1.21 – 1.56)	24.89, <0.001	1.17 (1.05 – 1.32)	7.41, 0.006

N number of complete observations used in the model; HL Hosmer-Lemeshow goodness of fit test, if $p>0.05$ then the model fits the data well; [a] people are assigned the OxCGRT value for stay-at-home orders that corresponds to the date they filled out the survey; [b] Wald Chi-square test from logistic regression with 1 degree of freedom comparing each level to the reference level within a variable, p-value based on Bonferroni correction; [c] OR is given for every 100 new cases reported. In instances where the confidence interval of the OR includes 1.00 yet is significant $p<0.05$, this is due to rounding error.

Table S5: Adjusted odds ratios from logistic regression for the prevalence of "somewhat/much worse" versus "unchanged/improved" in each COVID-19 health related outcome and stringency index

For each of the following, indicate how you have been personally affected by the COVID-19 pandemic (For each endpoint the probability of "Somewhat/much worse" is modelled, vs "unchanged/improved"). Odds ratio OR (95% confidence interval CI) is always compared to the reference category.

Variable	Categories	Physical health		Mental health		Stress in life		Overall wellbeing	
		**OR (95% CI)**	**χ^2_1, p-value [b]**	**OR (95% CI)**	**χ^2_1, p-value [b]**	**OR (95% CI)**	**χ^2_1, p-value [b]**	**OR (95% CI)**	**χ^2_1, p-value [b]**
N, HL p-value		5874, 0.20		5864, 0.01		5873, 0.36		5873, 0.35	
Stringency Index (SI) [a]	80≤SI	1.22 (0.90 - 1.65)	1.65, 0.199	1.30 (0.95 - 1.79)	2.65, 0.104	1.05 (0.76 - 1.46)	0.10, 0.756	1.39 (1.02 - 1.89)	4.44, 0.035
	70≤SI<80	0.96 (0.74 - 1.25)	0.09, 0.764	0.81 (0.62 - 1.06)	2.29, 0.130	0.67 (0.50 - 0.88)	8.27, 0.004	1.03 (0.79 - 1.34)	0.03, 0.852
	60≤SI<70	0.91 (0.68 - 1.21)	0.41, 0.521	1.10 (0.82 - 1.49)	0.42, 0.519	0.74 (0.54 – 1.00)	3.88, 0.049	1.09 (0.82 - 1.45)	0.34, 0.560
	50≤SI<60	Reference		Reference		Reference		Reference	
Age group	18 to 34	1.37 (1.16 - 1.63)	13.10, <0.001	1.72 (1.43 - 2.06)	33.89, <0.001	1.42 (1.18 - 1.70)	13.43, <0.001	1.26 (1.06 - 1.50)	6.75, 0.009
	35 to 54	1.25 (1.06 - 1.46)	7.44, 0.006	1.37 (1.16 - 1.61)	14.36, <0.001	1.21 (1.03 - 1.43)	5.16, 0.023	1.17 (1.00 – 1.37)	3.62, 0.057
	55+	Reference		Reference		Reference		Reference	
Gender	Other/Prefer not to say	1.21 (0.70 - 2.09)	0.45, 0.504	2.28 (1.02 - 5.08)	4.03, 0.045	0.89 (0.47 - 1.67)	0.14, 0.708	0.93 (0.51 - 1.68)	0.06, 0.809
	Female	0.87 (0.78 - 0.97)	5.83, 0.016	1.13 (1.01 - 1.27)	4.33, 0.037	1.32 (1.17 - 1.49)	20.62, <0.001	1.03 (0.92 - 1.15)	0.21, 0.650
	Male	Reference		Reference		Reference		Reference	
Indigenous status	Indigenous	1.19 (0.93 - 1.53)	1.94, 0.163	1.00 (0.76 - 1.31)	0.00, 0.979	1.04 (0.79 - 1.38)	0.08, 0.781	1.02 (0.79 - 1.33)	0.03, 0.871
	Non-Indigenous	Reference		Reference		Reference		Reference	
Geographical area	Urban	1.25 (1.07 - 1.47)	7.43, 0.006	1.10 (0.93 - 1.31)	1.29, 0.256	1.12 (0.94 - 1.33)	1.70, 0.192	1.19 (1.01 - 1.40)	4.21, 0.040
	Suburban	1.22 (1.04 - 1.42)	6.29, 0.012	1.11 (0.94 - 1.30)	1.60, 0.206	1.18 (1.01 - 1.39)	4.16, 0.041	1.16 (0.99 - 1.35)	3.42, 0.064
	Rural/remote	Reference		Reference		Reference		Reference	
Income	$150K or more	0.98 (0.80 - 1.19)	0.06, 0.804	1.27 (1.03 - 1.56)	4.90, 0.027	1.35 (1.09 - 1.66)	7.60, 0.006	1.29 (1.05 - 1.57)	6.08, 0.014
	$80K - <$150K	1.10 (0.93 - 1.29)	1.25, 0.264	1.19 (0.99 - 1.42)	3.61, 0.057	1.19 (1.00 - 1.42)	3.66, 0.056	1.14 (0.96 - 1.35)	2.29, 0.130

	$40K - <$80K	1.03 (0.87 - 1.22)	0.12, 0.730	1.05 (0.88 - 1.26)	0.31, 0.577	1.06 (0.89 - 1.27)	0.41, 0.523	0.99 (0.83 - 1.17)	0.03, 0.871
	less than $40K	Reference		Reference		Reference		Reference	
Work/attend school outside home	Yes	0.87 (0.70 - 1.07)	1.85, 0.174	1.07 (0.86 - 1.34)	0.36, 0.546	1.07 (0.85 - 1.34)	0.29, 0.591	1.00 (0.81 - 1.24)	0.00, 0.977
	No	Reference		Reference		Reference		Reference	
Work/attend school inside home	Yes	1.04 (0.85 - 1.27)	0.12, 0.724	0.98 (0.78 - 1.22)	0.05, 0.827	1.03 (0.82 - 1.29)	0.05, 0.815	0.96 (0.77 - 1.18)	0.16, 0.686
	No	Reference		Reference		Reference		Reference	
Retired	Yes	0.85 (0.66 - 1.10)	1.57, 0.210	0.75 (0.57 - 0.98)	4.57, 0.032	0.73 (0.56 - 0.96)	4.95, 0.026	0.75 (0.57 - 0.97)	4.85, 0.028
	No	Reference		Reference		Reference		Reference	
Unemployed	Yes	1.04 (0.80 - 1.36)	0.10, 0.752	1.09 (0.81 - 1.46)	0.31, 0.578	1.19 (0.88 - 1.61)	1.28, 0.257	1.05 (0.79 - 1.38)	0.10, 0.758
	No	Reference		Reference		Reference		Reference	
On paid leave (sick leave, maternity, disability)	Yes	0.62 (0.42 - 0.91)	6.00, 0.014	1.27 (0.81 - 1.98)	1.07, 0.301	1.02 (0.65 - 1.59)	0.00, 0.945	1.23 (0.81 - 1.89)	0.95, 0.330
	No	Reference		Reference		Reference		Reference	
Other	Yes	1.09 (0.78 - 1.55)	0.26, 0.607	1.23 (0.85 - 1.77)	1.17, 0.280	1.06 (0.73 - 1.54)	0.09, 0.763	0.89 (0.63 - 1.28)	0.37, 0.541
	No	Reference		Reference		Reference		Reference	
Rated physical health in general, for one's age	Fair/poor	1.77 (1.52 - 2.07)	53.17, <0.001	0.94 (0.79 - 1.11)	0.56, 0.454	1.28 (1.07 - 1.54)	7.36, 0.007	1.33 (1.12 - 1.57)	10.96, <0.001
	Excellent/very good/good	Reference		Reference		Reference		Reference	
Rated mental health in general, for one's age	Fair/poor	1.50 (1.28 - 1.75)	25.99, <0.001	3.47 (2.83 - 4.26)	143.48, <0.001	2.26 (1.85 - 2.76)	62.78, <0.001	2.62 (2.19 - 3.14)	111.86, <0.001
	Excellent/very good/good	Reference		Reference		Reference		Reference	
Anxiety or depression	Diagnosed	1.57 (1.34 - 1.84)	32.41, <0.001	1.99 (1.68 - 2.36)	63.07, <0.001	1.59 (1.34 - 1.89)	27.40, <0.001	1.96 (1.67 - 2.31)	66.20, <0.001
	Suffer	1.56 (1.35 - 1.80)	35.52, <0.001	2.87 (2.42 - 3.39)	152.44, <0.001	2.48 (2.08 - 2.95)	103.03, <0.001	2.43 (2.08 - 2.83)	126.53, <0.001
	Does not apply	Reference		Reference		Reference		Reference	
Rate of deaths during the previous 7 days		0.84 (0.64 - 1.11)	1.50, 0.220	0.87 (0.65- 1.17)	0.84, 0.359	0.59 (0.44 - 0.80)	11.78, <0.001	0.90 (0.68 - 1.20)	0.51, 0.475
Rate of cases during the previous 7 days [c]		1.26 (1.11 – 1.42)	13.47, <0.001	1.16 (1.02 – 1.32)	4.73, 0.030	1.42 (1.24 – 1.63)	25.19, <0.001	1.15 (1.01 – 1.31)	4.75, 0.029

N number of complete observations used in the model; HL Hosmer-Lemeshow goodness of fit test, if p>0.05 then the model fits the data well; [a] people are assigned the OxCGRT value for stringency index that corresponds to the date they filled out the survey; [b] Wald Chi-square test from

logistic regression with 1 degree of freedom comparing each level to the reference level within a variable, p-value based on Bonferroni correction; [c] OR is given for every 100 new cases reported.

Parameter estimates derived from variables having a coefficient of variation (CV) between 16.6% and 33.3% were designated “E” and must be interpreted with caution due to the high sampling variability associated with it. No results are reported for cell frequencies less than 10. In instances where the confidence interval of the OR includes 1.00 yet is significant $p<0.05$, this is due to rounding error.

Table S6: Prevalence (in percentage) of reported impact of COVID-19 on health-related outcomes and school closures

Reported impact of COVID-19 on health-related outcomes		School Closure value[a]		
		1 (n=1235)	2 (n=2464)	3 (n=2925)
Physical health	Somewhat/much worse	39.8 (37.1 - 42.5)	39.6 (37.7 - 41.6)	47.4 (45.6 - 49.2)
	Unchanged	46.6 (43.9 - 49.4)	48.1 (46.2 - 50.1)	40.9 (39.1 - 42.7)
	Somewhat/much improved	13.6 (11.8 - 15.6)	12.2 (11.0 - 13.6)	11.7 (10.6 - 12.9)
Mental health	Somewhat/much worse	60.0 (57.2 - 62.7)	53.9 (51.9 - 55.9)	63.8 (62.0 - 65.5)
	Unchanged	34.8 (32.2 - 37.5)	40.4 (38.4 - 42.3)	32.3 (30.6 - 34)
	Somewhat/much improved	5.1 (4.0 - 6.5)	5.7 (4.9 - 6.7)	3.9 (3.3 - 4.7)
Stress in life	Somewhat/much worse	65.9 (63.2 - 68.5)	62.8 (60.9 - 64.7)	70.3 (68.6 – 72.0)
	Unchanged	28.1 (25.6 - 30.6)	31.1 (29.3 - 32.9)	24.8 (23.2 - 26.4)
	Somewhat/much improved	6.1 (4.9 - 7.5)	6.1 (5.2 - 7.1)	4.9 (4.2 - 5.7)
Overall wellbeing	Somewhat/much worse	53.5 (50.7 - 56.3)	50.0 (48.0 – 52.0)	59.3 (57.5 – 61.0)
	Unchanged	38.1 (35.4 - 40.9)	38.6 (36.7 - 40.6)	32.5 (30.8 - 34.2)
	Somewhat/much improved	8.4 (7.0 - 10.1)	11.4 (10.2 - 12.7)	8.2 (7.3 - 9.3)

[a] Participants are assigned the OxCGRT value for school closures that corresponds to the date they filled out the survey. There were no participants at level 0 of school closures, School closures: value 3 – all levels of school closed; value 2 – some levels of school closed; value 1 – schools recommended to close.

Table S7: Prevalence (in percentage) of reported impact of COVID-19 on health-related outcomes and business/workplace closures

Reported impact of COVID-19 on health-related outcomes		Business/workplace closure value[a]			
		0 (n=115)	1 (n=119)	2 (n=3261)	3 (n=3129)
Physical health	Somewhat/much worse	32.6 (24.7 - 41.6)	36.1 (28.0 - 45.1)	39.8 (38.2 - 41.5)	47.2 (45.4 - 48.9)
	Unchanged	52.9 (43.8 - 61.8)	53.8 (44.8 - 62.5)	47.7 (46.0 - 49.4)	40.8 (39.1 - 42.5)
	Somewhat/much improved	14.5 (9.2 - 22.1) [E]	10.1 (5.9 - 16.8) [E]	12.5 (11.4 - 13.6)	12.0 (10.9 - 13.2)
Mental health	Somewhat/much worse	51.4 (42.4 - 60.4)	50.2 (41.3 – 59.0)	56.1 (54.4 - 57.8)	63.5 (61.8 - 65.1)
	Unchanged	44.7 (36.0 - 53.9)	43.6 (35.0 - 52.6)	38.3 (36.6 - 40)	32.5 (30.9 - 34.2)
	Somewhat/much improved	X	X	5.6 (4.9 - 6.4)	4.0 (3.4 - 4.7)
Stress in life	Somewhat/much worse	64.2 (55.1 - 72.4)	60.9 (51.9 - 69.2)	63.9 (62.2 - 65.5)	69.9 (68.3 - 71.5)
	Unchanged	30.3 (22.7 - 39.3)	29.7 (22.2 - 38.5)	30.0 (28.4 - 31.6)	25.2 (23.7 - 26.8)
	Somewhat/much improved	X	9.4 (5.3 – 16.0) [E]	6.1 (5.3 – 7.0)	4.8 (4.1 - 5.7)
Overall wellbeing	Somewhat/much worse	46.8 (37.9 - 55.9)	45.0 (36.3 – 54.0)	51.7 (50.0 - 53.4)	58.6 (56.8 - 60.3)
	Unchanged	47.2 (38.3 - 56.3)	46.4 (37.7 - 55.3)	37.6 (35.9 - 39.2)	33.2 (31.5 - 34.8)
	Somewhat/much improved	X	8.6 (4.8 – 15.0) [E]	10.7 (9.7 - 11.8)	8.2 (7.3 - 9.3)

[a] Participants are assigned the OxCGRT value for business/workplace closures that corresponds to the date they filled out the survey. Business/workplace closures: value 3 – all non-essential businesses/workplaces closed; value 2 – some non-essential businesses/workplaces closed; value 1 – recommend to close non-essential businesses/workplaces; value 0 – no closures.

X less than 10 observations in the cell; E coefficient of variation (noted as [E]) is between 16.6% and 33.3%, interpret with caution

Table S8: Prevalence (in percentage) of reported impact of COVID-19 on health-related outcomes and restrictions on gatherings

Reported impact of COVID-19 on health-related outcomes		Restrictions on gathering value [a]	
		3 (n=375)	4 (n=6249)
Physical health	Somewhat/much worse	39.2 (34.4 - 44.2)	43.3 (42.1 - 44.6)
	Unchanged	48.0 (42.9 – 53.0)	44.4 (43.2 - 45.7)
	Somewhat/much improved	12.8 (9.8 - 16.6)	12.2 (11.4 - 13.1)
Mental health	Somewhat/much worse	53.9 (48.8 - 58.9)	59.7 (58.5 - 60.9)
	Unchanged	41.3 (36.5 - 46.4)	35.4 (34.3 - 36.6)
	Somewhat/much improved	4.8 (3.0 - 7.4) [E]	4.8 (4.3 - 5.4)
Stress in life	Somewhat/much worse	61.6 (56.5 - 66.3)	67.0 (65.8 - 68.2)
	Unchanged	32.1 (27.5 – 37.0)	27.5 (26.4 - 28.6)
	Somewhat/much improved	6.4 (4.3 - 9.3) [E]	5.5 (5.0 - 6.1)
Overall wellbeing	Somewhat/much worse	48.6 (43.6 - 53.7)	55.1 (53.9 - 56.3)
	Unchanged	44.2 (39.2 - 49.2)	35.3 (34.1 - 36.5)
	Somewhat/much improved	7.2 (5.0 - 10.3) [E]	9.6 (8.9 - 10.3)

[a] Participants are assigned the OxCGRT value for restrictions on gatherings that corresponds to the date they filled out the survey. There were no participants at level 0, 1 and 2 of restrictions on gatherings, Restrictions on gatherings: value 4 – 10 people or less permitted to meet; value 3 – 11 to 100 people permitted to meet.

E coefficient of variation (noted as [E]) is between 16.6% and 33.3%, interpret with caution

Table S9: Prevalence (in percentage) of reported impact of COVID-19 on health-related outcomes and stay-at-home orders

Reported impact of COVID-19 on health-related outcomes		Stay-at-home orders value [a]	
		1 (n=2378)	2 (n=4246)
Physical health	Somewhat/much worse	42.4 (40.4 - 44.4)	43.5 (42.0 – 45.0)
	Unchanged	45.1 (43.1 - 47.1)	44.4 (42.9 - 45.9)
	Somewhat/much improved	12.5 (11.3 - 13.9)	12.1 (11.1 - 13.1)
Mental health	Somewhat/much worse	60.9 (58.9 - 62.9)	58.5 (57.1 – 60.0)
	Unchanged	33.9 (32 - 35.8)	36.8 (35.4 - 38.3)
	Somewhat/much improved	5.2 (4.4 - 6.2)	4.6 (4.0 - 5.3)
Stress in life	Somewhat/much worse	69.0 (67.1 - 70.8)	65.4 (64.0 - 66.8)
	Unchanged	25.9 (24.2 - 27.7)	28.8 (27.4 - 30.1)
	Somewhat/much improved	5.1 (4.3 - 6.1)	5.8 (5.1 - 6.5)
Overall wellbeing	Somewhat/much worse	54.9 (52.9 - 56.9)	54.7 (53.2 - 56.2)
	Unchanged	36.8 (34.9 - 38.7)	35.3 (33.9 - 36.7)
	Somewhat/much improved	8.3 (7.3 - 9.5)	10.1 (9.2 – 11.0)

[a] Participants are assigned the OxCGRT value for stay-at-home orders that corresponds to the date they filled out the survey. There were no participants at values 0 and 3 of stay-at-home orders, Stay-at-home orders: value 2 – leaving home permitted for essential trips; value 1 – recommend not leave home.

Table S10: Prevalence (in percentage) of reported impact of COVID-19 on health-related outcomes and stringency index

Reported impact of COVID-19 on health-related outcomes		Stringency index value[a]			
		50 - <60 (n=2714)	60 - <70 (n=1693)	70 - <80 (n=1867)	80+ (n=350)
Physical health	Somewhat/much worse	36.9 (32.0 - 42.1)	42.4 (40.2 - 44.7)	37.2 (35 - 39.6)	48.0 (46.2 - 49.9)
	Unchanged	52.4 (47.1 - 57.6)	44.5 (42.2 - 46.7)	50.9 (48.6 - 53.3)	39.8 (38 - 41.7)
	Somewhat/much improved	10.8 (7.9 - 14.4)	13.1 (11.7 - 14.7)	11.8 (10.4 - 13.4)	12.1 (10.9 - 13.4)
Mental health	Somewhat/much worse	54.9 (49.6 – 60.0)	61.5 (59.3 - 63.7)	50.6 (48.2 – 53.0)	64.0 (62.2 - 65.8)
	Unchanged	42.1 (37.0 - 47.3)	32.7 (30.6 - 34.9)	43.9 (41.6 - 46.3)	32.0 (30.3 - 33.8)
	Somewhat/much improved	3.0 (1.7 - 5.4) [E]	5.8 (4.8 - 6.9)	5.5 (4.5 - 6.7)	4.0 (3.3 - 4.8)
Stress in life	Somewhat/much worse	65.8 (60.7 - 70.6)	68.9 (66.7 - 70.9)	57.6 (55.2 - 59.9)	71.0 (69.3 - 72.7)
	Unchanged	29.3 (24.8 - 34.3)	25.8 (23.8 - 27.8)	35.8 (33.6 - 38.1)	23.8 (22.3 - 25.5)
	Somewhat/much improved	4.9 (3.1 - 7.7) [E]	5.4 (4.4 - 6.5)	6.6 (5.5 - 7.9)	5.2 (4.4 - 6.1)
Overall wellbeing	Somewhat/much worse	49.0 (43.8 - 54.2)	55.1 (52.8 - 57.3)	48.2 (45.8 - 50.6)	59.3 (57.5 - 61.2)
	Unchanged	45.2 (40.1 - 50.5)	35.9 (33.7 - 38.1)	39.7 (37.4 - 42.1)	32.1 (30.4 - 33.9)
	Somewhat/much improved	5.8 (3.8 - 8.8) [E]	9.1 (7.8 - 10.5)	12.1 (10.6 - 13.7)	8.5 (7.5 - 9.6)

[a] Participants are assigned the OxCGRT stringency index value that corresponds to the date they filled out the survey.

E coefficient of variation (noted as [E]) is between 16.6% and 33.3%, interpret with caution

Multinomial logistic regression model modelling the prevalence of "much worse" and "somewhat worse" compared to "unchanged/improved". The first table reports the odds ratios for the prevalence of "much worse" versus "unchanged / improved". The second table reports the odds ratios for the prevalence of "somewhat worse" versus "unchanged / improved". Tables S11 and S12 present the adjusted odds ratios extracted from the 20 multinomial models which are equivalent to the main models regarding the covariates. Odds ratios of the covariates are suppressed for ease of reading the results.

Table S11: Adjusted odds ratios for the prevalence of "much worse" versus "unchanged/improved" in the multinomial logistic regression model

OxCGRT Variable[a]	**Class**	**Physical health**		**Mental health**		**Stress in life**		**Overall wellbeing**	
		**OR (95% CI)**	**χ^2_1, p-value [b]**	**OR (95% CI)**	**χ^2_1, p-value [b]**	**OR (95% CI)**	**χ^2_1, p-value [b]**	**OR (95% CI)**	**χ^2_1, p-value [b]**
Stringency Index	80+	1.07 (0.59 - 1.94)	0.06, 0.814	1.48 (0.88 - 2.48)	2.21, 0.137	0.98 (0.62 - 1.56)	0.01, 0.934	1.07 (0.62 - 1.86)	0.06, 0.808
	70 to <80	0.95 (0.56 - 1.61)	0.04, 0.841	1.13 (0.71 - 1.79)	0.26, 0.610	0.75 (0.50 - 1.13)	1.88, 0.170	1.21 (0.74 - 1.98)	0.59, 0.441
	60 to <70	0.75 (0.43 - 1.33)	0.95, 0.330	1.01 (0.62 - 1.67)	0.00, 0.955	0.77 (0.49 - 1.20)	1.34, 0.246	0.96 (0.56 - 1.63)	0.03, 0.874
	50 to <60	Reference		Reference		Reference		Reference	
School closure	3=All levels of school closed	1.11 (0.75 - 1.64)	0.28, 0.599	1.40 (1.02 - 1.93)	4.21, 0.040	1.21 (0.90 - 1.62)	1.66, 0.197	1.01 (0.71 - 1.44)	0.00, 0.946
	2=Some levels of school closed	1.06 (0.77 - 1.46)	0.14, 0.710	0.91 (0.69 - 1.20)	0.44, 0.506	0.90 (0.71 - 1.15)	0.71, 0.400	0.98 (0.73 - 1.30)	0.03, 0.874
	1=Schools recommended to close	Reference		Reference		Reference		Reference	
	0=No restrictions	No data		No data		No data		No data	
Business/ workplace closure	3=All non-essential businesses/workplaces closed	0.78 (0.30 - 1.99)	0.27, 0.600	2.58 (1.05 - 6.33)	4.29, 0.038	0.85 (0.40 - 1.82)	0.17, 0.685	1.27 (0.52 - 3.14)	0.28, 0.598
	2=Some non-essential businesses/workplaces closed	0.65 (0.27 - 1.57)	0.92, 0.338	1.67 (0.71 - 3.93)	1.36, 0.244	0.66 (0.32 - 1.35)	1.30, 0.255	1.14 (0.49 - 2.68)	0.09, 0.759
	1=Recommend to close non-essential businesses/workplaces	1.30 (0.41 - 4.17)	0.20, 0.659	1.45 (0.45 - 4.63)	0.39, 0.532	0.57 (0.20 - 1.59)	1.15, 0.283	0.68 (0.19 - 2.43)	0.35, 0.553
	0=No closures	Reference		Reference		Reference		Reference	
Restrictions on gatherings	4 = ≤10 people	0.65 (0.38 - 1.13)	2.33, 0.127	0.88 (0.55 - 1.42)	0.27, 0.602	0.76 (0.50 - 1.16)	1.60, 0.206	1.04 (0.62 - 1.76)	0.03, 0.873
	3 = 11-100 people	Reference		Reference		Reference		Reference	
	2 = 101-1000 people	No data		No data		No data		No data	
	1 = >1000 people	No data		No data		No data		No data	
	0 = no restrictions	No data		No data		No data		No data	
Stay at home orders	3=Leaving home permitted with minimal exceptions	No data		No data		No data		No data	
	2=Leaving home permitted for essential trips	1.16 (0.81 - 1.67)	0.68, 0.410	1.00 (0.74 - 1.36)	0.00, 0.999	0.88 (0.67 - 1.16)	0.82, 0.365	1.31 (0.94 - 1.84)	2.51, 0.113

	1=Recommend not leave home	Reference		Reference		Reference		Reference	
	0=No restrictions	No data		No data		No data		No data	

[a] Participants are assigned the OxCGRT value for stringency index, school closures, business/workplace closures, restrictions on gatherings and stay-at-home orders that corresponds to the date they filled out the survey; [b] All models were adjusted for age, gender, Indigenous status, geographical location, income, work status, general physical and mental health, anxiety/depression, rate of death due to COVID-19 and rate of COVID-19 cases per 100,000. Wald Chi-square test from logistic regression with 1 degree of freedom comparing each level to the reference level within a variable, p-value based on Bonferroni correction.

Table S12: Adjusted odds ratios for the prevalence of "somewhat worse" versus "unchanged/improved" in the multinomial logistic regression model

OxCGRT Variable[a]	Class	Physical health		Mental health		Stress in life		Overall wellbeing	
		**OR (95% CI)**	**χ^2_1, p-value [b]**	**OR (95% CI)**	**χ^2_1, p-value [b]**	**OR (95% CI)**	**χ^2_1, p-value [b]**	**OR (95% CI)**	**χ^2_1, p-value [b]**
Stringency Index	80+	1.25 (0.91 - 1.71)	1.86, 0.172	1.27 (0.92 - 1.76)	2.14, 0.144	1.08 (0.78 - 1.51)	0.22, 0.638	1.45 (1.06 - 2.00)	5.37, 0.020
	70 to <80	0.96 (0.73 - 1.27)	0.08, 0.775	0.77 (0.58 - 1.01)	3.50, 0.061	0.65 (0.49 - 0.87)	8.42, 0.004	1.00 (0.76 - 1.31)	0.00, 0.998
	60 to <70	0.94 (0.70 - 1.27)	0.15, 0.695	1.14 (0.84 - 1.54)	0.67, 0.412	0.74 (0.54 - 1.01)	3.50, 0.061	1.12 (0.83 - 1.50)	0.55, 0.460
	50 to <60	Reference		Reference		Reference		Reference	
School closure	3=All levels of school closed	1.15 (0.94 - 1.40)	1.90, 0.168	1.00 (0.81 - 1.22)	0.00, 0.971	1.22 (0.99 - 1.50)	3.31, 0.069	1.19 (0.98 - 1.45)	2.95, 0.086
	2=Some levels of school closed	0.93 (0.79 - 1.10)	0.68, 0.410	0.67 (0.57 - 0.79)	21.24, <0.001	0.87 (0.73 - 1.03)	2.48, 0.115	0.82 (0.69 - 0.96)	5.79, 0.016
	1=Schools recommended to close	Reference		Reference		Reference		Reference	
	0=No restrictions	No data		No data		No data		No data	
Business/ workplace closure	3=All non-essential businesses/workplaces closed	1.47 (0.88 - 2.47)	2.12, 0.145	1.50 (0.91 - 2.49)	2.51, 0.113	0.81 (0.48 - 1.37)	0.61, 0.436	1.41 (0.86 - 2.33)	1.86, 0.172
	2=Some non-essential businesses/workplaces closed	1.15 (0.70 - 1.88)	0.29, 0.590	1.06 (0.66 - 1.70)	0.06, 0.811	0.59 (0.36 - 0.96)	4.46, 0.035	1.04 (0.65 - 1.67)	0.03, 0.856
	1=Recommend to close non-essential businesses/workplaces	1.54 (0.82 - 2.91)	1.79, 0.181	1.27 (0.68 - 2.36)	0.57, 0.450	0.85 (0.45 - 1.59)	0.27, 0.604	1.09 (0.59 - 2.01)	0.08, 0.784
	0=No closures	Reference		Reference		Reference		Reference	
Restrictions on gatherings	4 = ≤10 people	0.71 (0.54 - 0.94)	5.88, 0.015	0.83 (0.63 - 1.11)	1.57, 0.211	0.76 (0.57 - 1.01)	3.59, 0.058	0.88 (0.66 - 1.16)	0.86, 0.353
	3 = 11-100 people	Reference		Reference		Reference		Reference	
	2 = 101-1000 people	No data		No data		No data		No data	
	1 = >1000 people	No data		No data		No data		No data	
	0 = no restrictions	No data		No data		No data		No data	
Stay at home orders	3= Leaving home permitted for minimal exceptions	No data		No data		No data		No data	
	2=Leaving home permitted for essential trips	0.97 (0.81 - 1.17)	0.08, 0.772	0.71 (0.59 - 0.87)	11.54, <0.001	0.79 (0.65 - 0.96)	5.49, 0.019	0.88 (0.73 - 1.06)	1.76, 0.185
	1=Recommend not leave home	Reference		Reference		Reference		Reference	
	0=No restrictions	No data		No data		No data		No data	

[a] Participants are assigned the OxCGRT value for stringency index, school closures, business/workplace closures, restrictions on gatherings and stay-at-home orders that corresponds to the date they filled out the survey; [b] All models were adjusted for age, gender, Indigenous status,

geographical location, income, work status, general physical and mental health, anxiety/depression, rate of death due to COVID-19 and rate of COVID-19 cases per 100,000. Wald Chi-square test from logistic regression with 1 degree of freedom comparing each level to the reference level within a variable, p-value based on Bonferroni correction.
